# Non-canonical Wnt signaling promotes directed migration of intestinal stem cells to sites of injury

**DOI:** 10.1038/s41467-021-27384-4

**Published:** 2021-12-09

**Authors:** Daniel Jun-Kit Hu, Jina Yun, Justin Elstrott, Heinrich Jasper

**Affiliations:** Genentech Inc., 1 DNA Way, South San Francisco, CA 94080 USA

**Keywords:** Regeneration, Adult stem cells, Time-lapse imaging, Cell migration, Cell signalling

## Abstract

Tissue regeneration after injury requires coordinated regulation of stem cell activation, division, and daughter cell differentiation, processes that are increasingly well understood in many regenerating tissues. How accurate stem cell positioning and localized integration of new cells into the damaged epithelium are achieved, however, remains unclear. Here, we show that enteroendocrine cells coordinate stem cell migration towards a wound in the *Drosophila* intestinal epithelium. In response to injury, enteroendocrine cells release the N-terminal domain of the PTK7 orthologue, Otk, which activates non-canonical Wnt signaling in intestinal stem cells, promoting actin-based protrusion formation and stem cell migration towards a wound. We find that this migratory behavior is closely linked to proliferation, and that it is required for efficient tissue repair during injury. Our findings highlight the role of non-canonical Wnt signaling in regeneration of the intestinal epithelium, and identify enteroendocrine cell-released ligands as critical coordinators of intestinal stem cell migration.

## Abstract (web summary)

Stem cell migration is critical during adult tissue regeneration. Here, the authors demonstrate that enteroendocrine cells coordinate stem cell migration towards sites of injury in the Drosophila intestine by activating non-canonical Wnt signaling.

## Introduction

The migration of somatic stem cells (SCs) has been described as an important phenomenon during the regeneration of various tissues^1–3^. Skin SCs, for example, undergo directed migration from the hair follicle to the damaged epidermis during wound healing^4–6^. Hematopoietic and mesenchymal SCs, in turn, are even capable of long-range movement, migrating from the bone marrow to inflamed tissues as part of the immune response^7–10^. The underlying mechanisms regulating and coordinating SC migration, however, have only recently been explored, and technical challenges in imaging living tissue have limited the ability to observe and study this process.

The adult *Drosophila* intestine serves as a powerful model to study intestinal stem cell (ISC) activity and function while, critically, enabling a live imaging platform to directly observe SC behavior in a barrier epithelium^11–16^. ISCs line the pseudo-stratified epithelium and give rise to all the other cell types of the intestine: enteroblasts (EBs, post-mitotic precursor cells), enterocytes (ECs, differentiated cells with scaffolding and nutrient absorption roles), and enteroendocrine cells (EEs, differentiated cells with secretory roles)^13,17^. ISCs are largely quiescent during homeostasis, but are activated to divide in response to tissue damage or during tissue growth^11,18,19^. Studies of ISC dynamics during regeneration have largely been constrained to static analysis of fixed tissue, but recent innovations in imaging live, wholemount intestinal explants has expanded the ability to investigate these processes in real-time, providing insights into symmetric and asymmetric ISC divisions, intracellular calcium signaling, cell loss, and cell fate determination and differentiation in the ISC lineage^11–16^.

Cell migration is an actin-based process in which members of the Rho family of GTPases establish polarity at the leading edge by activating the Arp2/3 complex and mDia^20–23^. These proteins, in turn, polymerize actin to form lamellipodia and filopodia, directing forward motion through forces generated from actin flow and actomyosin contractility^24–26^. During development and morphogenesis, non-canonical Wnt signaling links extracellular cues to actin rearrangement through the interaction of Wnt ligands with the cell surface receptors Frizzled (Fz), Ptk7 (Otk in *Drosophila*), and Ror2 (Ror in *Drosophila*)^27–33^. This interaction activates intracellular Disheveled (Dsh in *Drosophila*, Dvl in mammals), which leads to downstream activation of Rho GTPases and actin rearrangement^34–36^. The extent to which this pathway plays a role in adult SC migration and regeneration remains unresolved.

Here, we demonstrate that *Drosophila* ISCs rapidly initiate migration after enteropathogen infection and after localized tissue damage by laser ablation. This process is mediated by a signaling cascade relying on matrix-metalloproteinase (MMP) induction and Otk expression in EEs at the wound site, which in turn activates non-canonical Wnt signaling in ISCs, promoting the actin-dependent formation of lamellipodia, and migration of ISCs to the wound area. Impairing ISC migration hinders ISC proliferation as well as effective intestinal regeneration following tissue damage, and sensitizes animals to death by enteropathogen infection. We propose that MMP-mediated cleavage of Otk in EEs at the wound is a critical signal promoting ISC migration toward the site of epithelial injury, ensuring efficient regeneration.

## Results

### ISCs exhibit migratory behavior after tissue damage

**Fig. 1:**
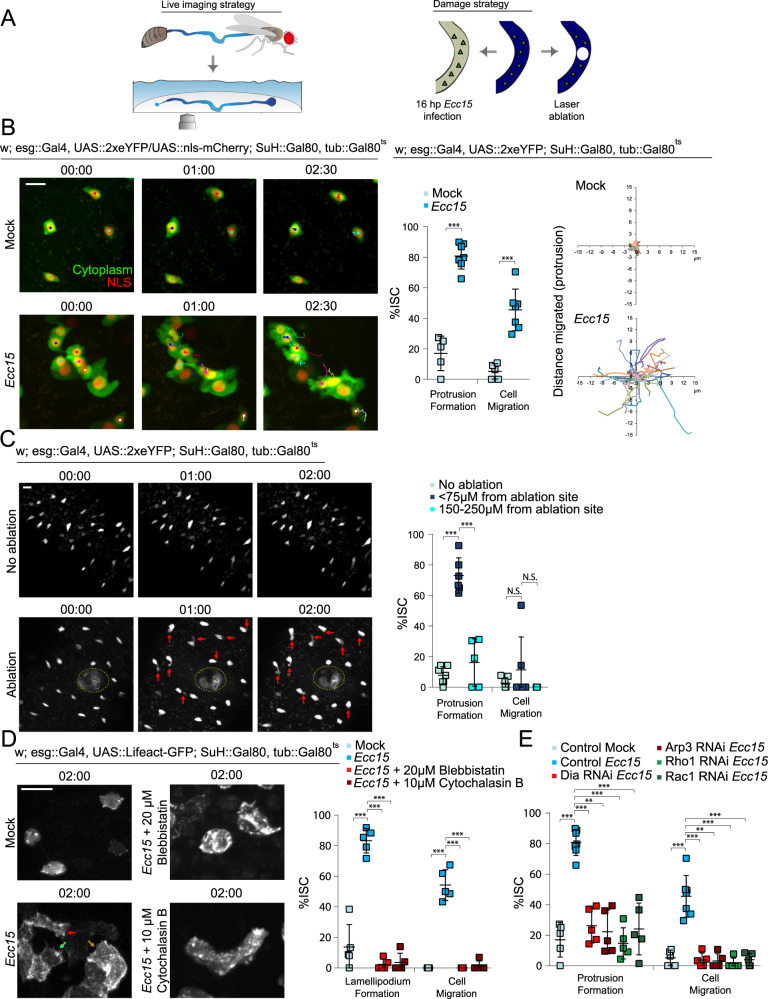
ISCs undergo migration following tissue damage. **A** Diagram representing ex vivo live imaging and damage strategy of *Drosophila* intestine. **B** Montage and quantification of protrusion formation and migration of ISCs in mock-treated and *Ecc15*-infected intestines. Montage tracks measured from the center of the nucleus, with starting x,y coordinate of each ISC normalized to 0,0. **C** Montage and quantification of protrusion formation and migration of ISCs in the undamaged intestine, near the wound (≤75 µm) of laser-ablated intestines, and far away from the wound (150–250 µm) of laser-ablated intestines. Red arrows indicate protrusions and the dotted line indicates ablation site. **D** ISCs formed lamellipodia after *Ecc15* infection. Lamellipodia formation and migration of ISCs dramatically decreased after inhibition of actomyosin function with small molecule inhibitors. Arrows indicate lamellipodia. **E** Expression of RNAi for proteins involved in regulating actin assembly decreased protrusion formation and migration of ISCs. Mock-treated and *Ecc15*-infected controls were taken from Fig. 1B, as they served as genetic controls and were performed in parallel. mean ± SD; *n* = sample size as follows, **B** 5 (mock) and 7 (*Ecc15)* flies from five independent experiments, **C**: 7 (no ablation), 6 (<75 µm) and 5 (150–250 µm) flies from 7, 6, and 5 independent experiments respectively, **D**: 5 flies from 2 independent experiments, **E**: 5 (all conditions except Control *Ecc15*) and 7 (Control *Ecc15*) flies from 5 (Control Mock, Control *Ecc15*), 2 (Dia RNAi, Rho1 RNAi, Rac1 RNAi), and 3 (Arp3 RNAi) independent experiments; N.S. = not significant (**C**: no ablation vs <75 µm = 0.3802, <75 µm vs 150–250 µm = 0.2665), ***P < 0.001 (**B**–**E** < 0.0001), based on two-tailed Student’s *t* test (**B**) and one-way ANOVA with Dunnett test (**C**–**E**). Scale bar = 10 µm. Timestamp indicated as hours:minutes. Source data are provided as a Source Data file. See also Supplementary Figs. 1–3, Supplementary Movies 1–11.

To visualize ISC behavior in response to damage, we imaged live, wholemount fly intestines ex vivo (Fig. 1A), a system we have previously utilized to directly observe ISC mitoses, EB differentiation, and intracellular calcium dynamics^11,12^. ISCs were identified by specific and inducible expression of cytoplasmic eYFP using the ISC/EB driver *escargot::Gal4* in combination with EB-specific expression of the Gal4 inhibitor Gal80 (Su(H)::Gal80), and ubiquitous expression of temperature-sensitive Gal80 (tub::Gal80^ts^)^37,38^. Flies were infected with *Erwinia carotovora carotovora 15* (*Ecc15*), a gram-negative bacterium that damages differentiated enterocytes, to induce a regenerative response (Fig. 1A)^39,40^. Under homeostatic conditions, ISCs were largely immotile, with little change in morphology (Fig. 1B, Supplementary Fig. 1a, and Supplementary Movie 1). In stark contrast, after 16 h post-*Ecc15* infection, ISCs were far more dynamic (Fig. 1B, Supplementary Fig. 1a, and Supplementary Movie 2). Over the 2.5 hr timelapse, ~80% of ISCs formed protrusions resembling a leading edge, indicative of migratory behavior. 57% of the ISCs that formed these protrusions fully migrated, as determined by translocation of the cell body of at least 3 µm. The migratory capability of ISCs in mock-treated or infected intestines was also compared by tracking the location of the cell body and the tip of the protrusion, or the most distal region of the cell cortex if a protrusion did not form (Fig. 1B and Supplementary Fig. 1a). When the x,y coordinates of protrusions or cell bodies at each time point were plotted on a Cartesian plane, both the cell body and the most distal part of the cell cortex traveled dramatically farther in ISCs from infected intestines than in controls. We asked whether other cell types were also capable of migration, and used the Su(H) and prospero promoters to identify post-mitotic precursor EBs and differentiated EEs, respectively. Only minimal protrusions and movement were observed in these cell types after *Ecc15* infection (Supplementary Fig. 1b and Supplementary Movies 3–4) suggesting that, in contrast to ISCs, more differentiated cells are integrated statically into the epithelium.

*Ecc15* causes widespread damage in the intestinal epithelium, and this paradigm was thus not suited for an examination of directed migration of ISCs. To generate a more localized epithelial wound instead, we utilized high-powered lasers with two-photon microscopy. In addition to creating a localized ~30 µm wound (Fig. 1C), this strategy also enables observation of ISC behavior immediately after damage. 73% of ISCs within 75 µm of the wound formed protrusions, compared with 8% in undamaged tissue (Fig. 1C, Supplementary Fig. 1c, and Supplementary Movie 5–6). This response was dependent on proximity to the wound as the majority of ISCs located farther away from the wound (150–250 µm) did not form protrusions (Fig. 1C).

Despite forming protrusions, the cell bodies of most ISCs within 75 µm of the wound did not translocate in the first 2.5 h post-ablation, potentially because the migratory response continues beyond this timeframe (the structural integrity of the ablated tissue began to deteriorate after 3 hours of live imaging, making longer imaging durations challenging). Nonetheless, we observed that the protrusions of ~81% of ISCs formed towards the wound, suggesting that migratory behavior was polarized towards the site of injury (Supplementary Fig. 2a). To assay for directionality of migration at later time points while avoiding potential toxicity from frequent two-photon microscopy, we imaged ablated guts immediately after ablation and 4.5 h later. We observed that most ISCs were located closer to the wound after 4.5 h whereas ISCs from unablated tissue rarely changed their position (Supplementary Fig. 2b). Furthermore, when damaged guts were cultured for 4.5 h before fixation, we observed that ISCs had accumulated around the periphery of the wound, and that there was a greater number of ISCs in a 40 µm radius around the ablation site than within the same area of the contralateral side of the intestine (Supplementary Fig. 2c; there was approximately the same number of ISCs on contralateral sides of undamaged intestines). Importantly, we did not observe any mitoses within the first 2.5 h after ablation (duration of live experiments) or at 4.5 h post-ablation (fixed experiments), suggesting that the accumulation of ISCs around the wound was a result of directed migration rather than the generation of additional ISCs by mitosis.

### ISC migration is dependent on actin-based lamellipodia

The presence of protrusions in migratory ISCs suggests that migration is driven by lamellipodia formation. To directly visualize whether a lamellipodium did indeed form, GFP-labeled LifeAct^14^ was expressed in ISCs. Under homeostatic conditions, cortical actin was observed around the periphery of ISCs (Fig. 1D and Supplementary Movie 7). After *Ecc15* infection, actin becomes enriched at the leading edge, and the formation of fliopodia and the lamellipodium were observed (Fig. 1D, Supplementary Fig. 3a, and Supplementary Movie 8). Following the extension of the lamellipodium, translocation of the cell body and retraction of the trailing edge then occurred. There were zero observed cases of translocation of the cell body without lamellipodium formation. To confirm that lamellipodia formation was actin-dependent, we utilized small molecule inhibitors to block actin polymerization or actomyosin contraction. The addition of either Cytochalasin B or Blebbistatin (small molecule inhibitors for actin network assembly and myosin II function respectively)^41,42^ abolished lamellipodia dynamics and ISC migration (Fig. 1E and Supplementary Movie 9). To further test the role of the lamellipodium in ISC migration, we examined proteins involved in regulating actin dynamics, and thus in lamellipodium formation. We tested the role of Dia (mDia1 in mammals) and the Arp2/3 complex, which promote actin nucleation and branching, as well as their upstream regulators, the GTPases Rho1 (RhoA in mammals) and Rac1^20,43^. Knocking down Dia, Arp3, Rho1, or Rac1 in ISCs of *Ecc15*-infected flies resulted in a significant decrease in protrusion formation and ISC migration compared to control flies (Fig. 1E, Supplementary Fig. 3b, and Supplementary Movie 10).

To better understand the mechanism(s) promoting translocation of the cell body, specifically the nucleus, we investigated the roles of nesprins in facilitating nuclear movement. Nesprins are part of the LINC complex that tether the cytoskeleton to the nuclear envelope, enabling nuclear positioning^44^. We depleted Klarsicht (Klar), a *Drosophila* nesprin, in ISCs from infected intestines, and found that the percentage of cell bodies that remained stationary increased compared to control flies (Supplementary Fig. 3c and 4b, and Supplementary Movie 11). Despite an inability to translocate the cell body, many of the ISCs after Klar depletion were still capable of forming protrusions, exhibiting a more elongated morphology as the leading edge often continued to extend. A Klar-mediated connection of the nucleus to the cytoskeleton is thus a critical component of the machinery executing ISC migration.

Altogether, these data support the idea that, in response to tissue damage, ISCs exhibit lamellipodia-dependent migration. Because ISCs are largely immotile under homeostatic conditions, ISC migration is likely part of the regenerative response to damage. To test this hypothesis, we sought to identify signaling mechanisms that may mediate the damage-induced activation of lamellipodia formation. Due to its role in cytoskeletal rearrangements and cell migration during development, we focused on the non-canonical Wnt signaling pathway^34,36^.

### Non-canonical Wnt signaling promotes ISC migration

**Fig. 2:**
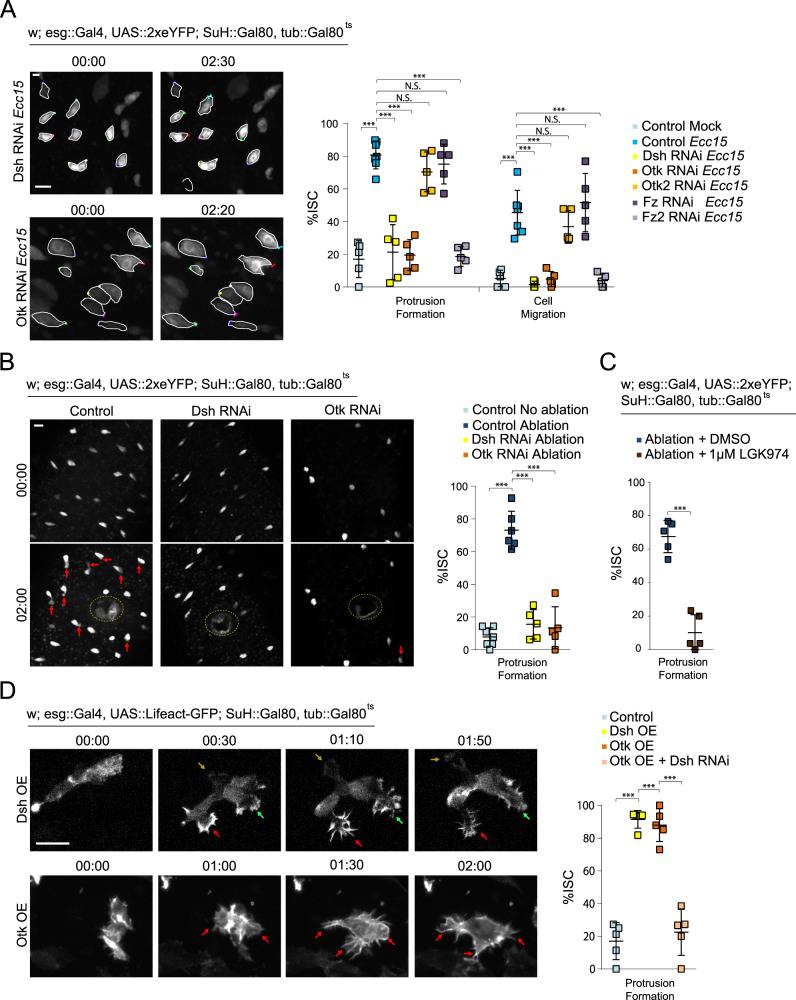
Non-canonical Wnt activity promotes ISC migration. **A** Quantification of ISC migratory behavior after genetic perturbation, and montage after depleting Dsh and Otk. ISCs are outlined in white, and montage tracks are measured from the most distal region of the cell cortex. Mock-treated and *Ecc15*-infected controls were taken from Fig. 1B, as they served as genetic controls and were performed in parallel. **B** Montage and quantification of ISC protrusion formation after laser ablation in Dsh- and Otk-depleted intestines. Red arrows indicate protrusions and the dotted line indicates the ablation site. Undamaged and ablated controls were taken from Fig. 1C, as they served as genetic controls. **C** Quantification of ISC protrusion formation in ablated intestines after inhibiting Wnt secretion versus DMSO-treated controls. **D** Montage and quantification of lamellipodia formation after overexpressing (OE) Dsh or Otk. Lamellipodia formation induced by Otk overexpression is suppressed after depleting Dsh. Arrows indicate protrusions. mean ± SD; *n* = sample size as follows, **A**: 5 (all conditions except Control *Ecc15*) and 7 (Control *Ecc15*) flies from 5 (Control Mock, Control *Ecc15*), 2 (Dsh RNAi, Otk RNAi, Fz RNAi, Fz2 RNAi), and 3 (Otk2 RNAi) independent experiments, **B**: 7 (no ablation), 6 (ablation), and 5 (Dsh RNAi, Otk RNAi) flies from 7 (no ablation), 6 (ablation), and 5 (Dsh RNAi, Otk RNAi) independent experiments, **C**: 5 flies from 5 independent experiments, **D**: 5 flies from 5 (Control), 2 (Dsh OE, Otk OE + Dsh RNAi), and 3 (Otk OE) independent experiments. N.S. = not significant (**A** – Protrusion Formation: *Ecc15* vs Otk2 RNAi = 0.4328, *Ecc15* vs Fz1 RNAi = 0.9012, **A** – Cell Migration: *Ecc15* vs Otk2 RNAi = 0.5092, *Ecc15* vs Fz1 RNAi = 0.7901), ***P < 0.001 (**A**–**D** < 0.0001), based on one-way ANOVA with Dunnett test (**A**, **B**), one-way ANOVA with Tukey test (**D**), and two-tailed Student’s *t* test (**C**). Scale bar = 10 µm. Timestamp indicated as hours:minutes. Source data are provided as a Source Data file. See also Supplementary Fig. 4, Supplementary Movies 12–17.

To test whether non-canonical Wnt signaling is required for ISC migration, we knocked down components of the pathway in ISCs, before infecting flies with *Ecc15*. Infection-induced cellular protrusions, as well as ISC migration, were no longer observed after depleting Dsh, Otk, or the second *Drosophila* Fz, Fz2, while knockdown of the second Drosophila Ptk7 orthologue, Otk2, or Fz had no effect (Fig. 2A, Supplementary Fig. 4a, b, and Supplementary Movie 12–13). Non-canonical Wnt signaling is also required for protrusion formation after laser injury, as knockdown of Dsh or Otk reduced protrusion formation (Fig. 2B and Supplementary Movie 14–15). Depleting Dsh or Otk in ablated tissue resulted in a loss of ISC accumulation around the wound at 4.5 h post-ablation, further supporting a role for non-canonical Wnt signaling during directed migration (Supplementary Fig. 4c). Protrusion formation was also reduced after treatment with the porcupine inhibitor LGK974^45^, which inhibits the release of Wnt ligands, indicating that secreted Wnt ligands play a critical role in inducing or maintaining migration of ISCs (Fig. 2C and Supplementary Movie 16). Wingless (Wg), a *Drosophila* Wnt, has been previously reported to be secreted from enteroblasts following damage of the *Drosophila* intestine, acting as an important regulation of ISC proliferation during tissue regeneration^46^. We tested whether EBs could also serve as a source of Wg to promote ISC migration after tissue damage and observed that, indeed, depleting Wg in EBs reduced the percentage of ISCs exhibiting migratory behavior (Supplementary Fig. 4d).

Activation of non-canonical Wnt signaling is also sufficient for the induction of migratory behavior in ISCs, as overexpressing either myc-tagged Dsh or GFP-tagged Otk in ISCs in unchallenged, undamaged guts caused dramatic rearrangement of the actin cytoskeleton (Fig. 2D and Supplementary Movie 17). The Otk-GFP signal is too weak to observe without an anti-GFP antibody, and is undetectable compared to the brighter GFP-tagged LifeAct. ISCs continually formed filopodia, often with multiple, seemingly non-polarized protrusions, which extended from different regions around the cell cortex. We confirmed that Dsh acts downstream of Otk in this context, as knockdown of Dsh while overexpressing Otk in ISCs impaired protrusion formation (Fig. 2D). Importantly, canonical Wnt signaling appears not to be involved in ISC migration, as knockdown of the canonical Wnt pathway transducers Armadillo (Arm; β-catenin in mammals) or Pangolin (Pan; TCF in mammals)^47,48^ did not affect ISC migration after *Ecc15* infection (Supplementary Fig. 4e).

**Fig. 3:**
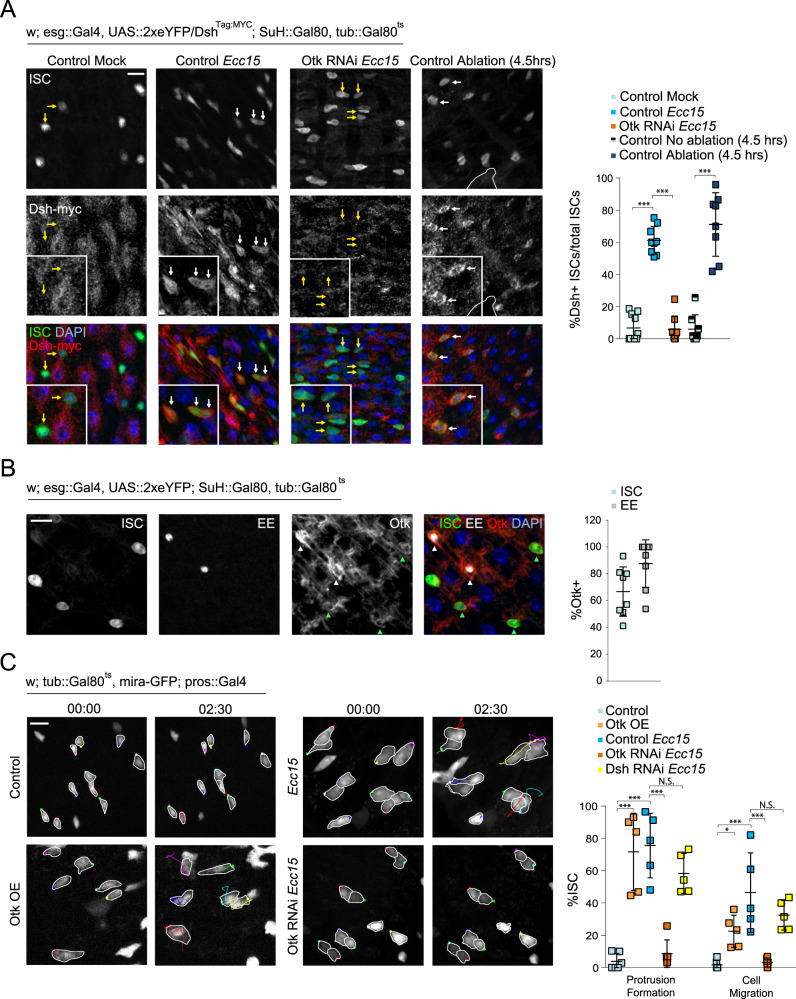
Localization of non-canonical Wnt pathway proteins, and role of EE-derived Otk in ISC migration. **A** Staining for endogenously myc-tagged Dsh reveals cortical decoration in ISCs after tissue damage, but absent or sparse localization in ISCs from undamaged tissue. Cortical Dsh is lost after Otk depletion in ISCs of damaged tissue. White outline indicates ablation site. White arrows indicate Dsh+ ISCs and yellow arrows indicate Dsh- ISCs. **B** Staining for Otk reveals expression predominantly in ISCs (green arrowhead) and EEs (white arrowhead). C. Montage and quantification of ISC migratory behavior after modulating Otk and Dsh expression in EEs. OE = overexpression. ISCs outlined in white, and montage tracks measured from the leading edge or, in cells that did not form protrusions, the most distal region of the cell cortex. mean ± SD; *n* = sample size as follows, **A**: 10 flies (Control Mock), 9 (Control *Ecc15* and Otk RNAi), and 8 (no ablation and ablation) flies from 2 (Control Mock, Control *Ecc15*, and Otk RNAi) and 3 (no ablation and ablation) independent experiments, **B**: 8 flies from 2 independent experiments, **C**: 6 (Control, Otk RNAi) and 5 flies (Otk OE, Control *Ecc15*, Dsh RNAi) from two (Control, Control *Ecc15*, Dsh RNAi,) and three (Otk OE, Otk RNAi) independent experiments; N.S. = not significant (**C** – protrusion formation: control *Ecc15* vs Dsh RNAi = 0.3908, **C** – cell migration: Control *Ecc15* vs Dsh RNAi = 0.3839), *P < 0.05 (**C** = 0.0361), ***P < 0.001 (**A**, **C** < 0.0001), based on one-way ANOVA with Tukey test. Scale bar = 10 µm. Timestamp indicated as hours:minutes. Source data are provided as a Source Data file. See also Supplementary Fig. 5, Supplementary Movies 18–19.

To further confirm and explore the role of non-canonical Wnt signaling in promoting ISC migration after damage, we assessed the protein expression and localization of Dsh and Otk. Dsh localization to the cell cortex is a hallmark of non-canonical Wnt signaling activity^49–51^. Using a fly line expressing myc-tagged Dsh under control of its endogenous promoter^52^, we found that Dsh was undetectable in ISCs from mock-treated, undamaged intestines, potentially either due to low expression or sparse localization at the cell membrane (Fig. 3A). Strikingly, after damage from either 16 h post *Ecc15* infection, or 1 or 4.5 h post laser ablation, Dsh became strongly localized to the cell membrane of ISCs, indicating activation of non-canonical Wnt signaling after damage (Fig. 3A and Supplementary Fig. 5a). Consistent with the role of Otk in this activation, Otk depletion in damaged intestines was sufficient to abolish Dsh localization to the ISC membrane (Fig. 3A).

To examine whether Otk could be detected at the cell membrane of ISCs, we generated antibodies recognizing the intracellular, C-terminal region of Otk, which successfully detected overexpressed GFP-tagged Otk at the cell membrane (Supplementary Fig. 5b). Otk was not detected in EB or EC cells, but was present in both ISCs and EEs (Fig. 3B). EEs were previously reported to play important roles in facilitating ISC activity^53^, and we tested whether EEs could also regulate ISC migration through Otk. Overexpression of Otk in EEs using the EE-specific driver prospero::Gal4, promoted protrusion formation in ISCs (detected in this experiment using miranda::GFP^54^), suggesting a non-autonomous role of Otk (Fig. 3C and Supplementary Movie 18). This effect seemed to be EE specific as overexpression of Otk in the EBs or ECs did not affect ISC migratory rates (Supplementary Fig. 5c). Conversely, depleting Otk in EE cells of *Ecc15*-infected guts was sufficient to decrease protrusion formation in ISCs, and impair ISC migration (Fig. 3C and Supplementary Movie 19). However, the role of EE-derived Otk in ISC migration did not seem to involve non-canonical Wnt signaling within EEs themselves as EE-specific depletion of Dsh did not affect ISC migration (Fig. 3C and Supplementary Fig. 5d). Because the Otk antibody also seemed to stain the muscle layer (Fig. 3B), we tested whether depleting Otk from the muscle layer had any effect on ISC migration, and found no impairment of migratory behavior (Supplementary Fig. 5e).

### EE-derived shedding of Otk is required for ISC migration

**Fig. 4:**
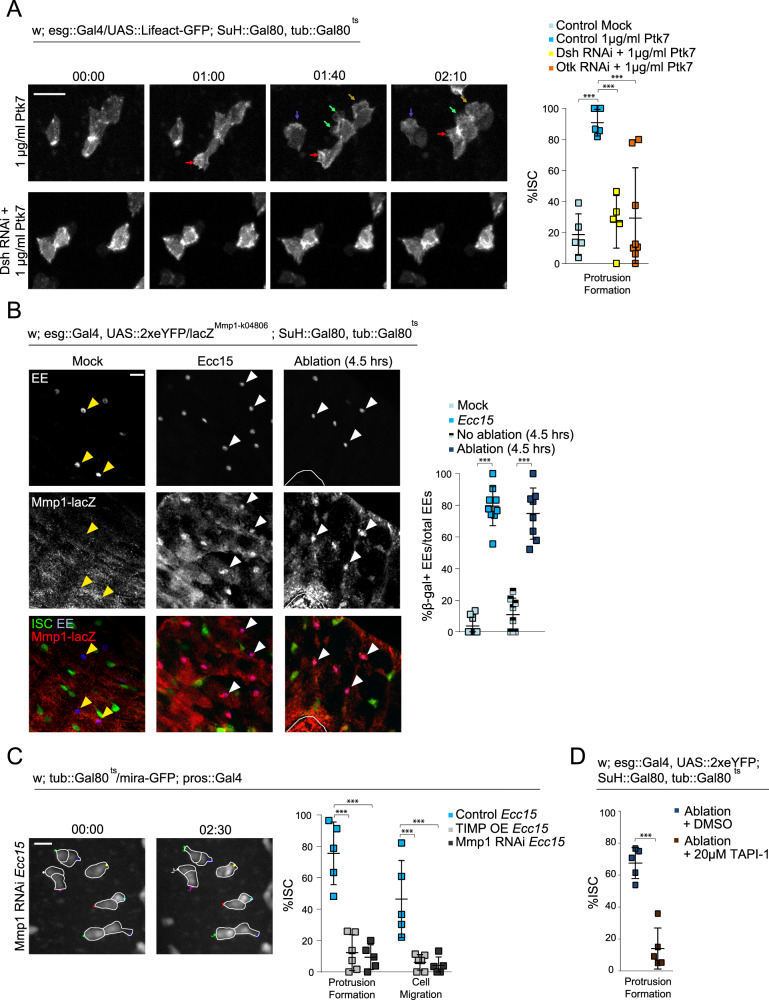
Injury-induced Mmp1 in EEs mediates ISC migration. **A** Montage and quantification of actin dynamics in ISCs after Ptk7 treatment in control, Dsh^RNAi^, and Otk^RNAi^ intestines. Arrows indicate protrusions. **B** Staining for beta Galactosidase in Mmp1-lacZ reporter lines reveals Mmp1 expression in EEs after tissue damage. White arrowheads indicate β-gal+ EEs and yellow arrowheads indicate β-gal-EEs. White outline indicates ablation site. **C** Montage and quantification of ISC migratory behavior after disrupting Mmp1 activity. OE = overexpression. *Ecc15*-infected controls were taken from Fig. 3C, as experiments were performed in parallel. ISCs are outlined in white, and montage tracks measured from the most distal region of the cell cortex. **D** Quantification of ISC protrusion formation in ablated intestines after inhibiting MMP activity versus DMSO-treated controls. DMSO-treated controls taken from Fig. 2C. mean ± SD; *n* = sample size as follows, **A**: 5 (all conditions except Otk RNAi + Ptk7) and 7 (Otk RNAi + Ptk7) flies from 3 (Control Mock, Otk RNAi) and 2 (Control Ptk7, Dsh RNAi) independent experiments, **B**: 9 (Mock, *Ecc15*) and 8 (no ablation, ablation) flies from 2 independent experiments, **C**: 5 (Control, Mmp1 RNAi) and 6 (Timp OE) flies from 2 independent experiments, **D**: 5 flies from 5 independent experiments; ***P < 0.001 (**A**: Control Mock vs Control Ptk7 = 0.0002, Control Ptk7 vs Dsh RNAi = 0.0007, Control Ptk7 vs Otk RNAi: 0.0004, **B**–**D** < 0.0001), based on one-way ANOVA with Dunnett test (**A**, **C**) and two-tailed Student’s *t* test (**B**, **D**). Scale bar = 10 µm. Timestamp indicated as hours:minutes. Source data are provided as a Source Data file. See also Supplementary Fig. 6, Supplementary Movies 20–23.

Previous studies in mammals have found that the extracellular, N-terminal region of Ptk7 can be cleaved from the cell membrane by MMP and ADAM metalloproteinases to promote migration in cancer cells^55–57^. We examined whether this “shed” form of Ptk7 was sufficient to induce migratory behavior in ISCs. Undamaged intestines were incubated with a recombinant, extracellular fragment of mouse Ptk7 (Met1-Glu683). Within an hour post incubation, the majority of ISCs underwent rapid filopodia formation with highly dynamic protrusions, phenocopying Otk overexpression (Fig. 4A and Supplementary Movie 20). The effect of extracellular Ptk7 was also dependent on non-canonical Wnt signaling in ISCs, as depleting Otk or Dsh in ISCs reduces protrusion formation (Fig. 4A and Supplementary Movie 21).

Because extracellular Ptk7 was sufficient to drive migratory behavior, we hypothesized that, in response to damage, Otk in EEs may be cleaved by MMPs to promote ISC migration. To test this idea, we assessed Mmp1 expression using an Mmp1-lacZ reporter line^58^. While beta Galactosidase expression was largely absent in EEs from undamaged tissue, beta Galactosidase expression increased strongly and specifically in EEs after *Ecc15* infection or laser ablation (Fig. 4B and Supplementary Fig 6a). We also used antibodies against Mmp1 to detect Mmp1 expression in EEs. Immunofluorescence with this antibody detects signals in the basal region of the epithelium of uninjured intestines but shows the minimal signal in EEs. After bacterial infection, however, Mmp1 staining in EEs increases, supporting the notion that Mmp1 expression is increased in EEs after injury (Supplementary Fig. 6b). We inhibited Mmp1 activity specifically in EEs by overexpression of its inhibitor, TIMP, or by depletion of Mmp1, and observed a drastic reduction of protrusion formation and migratory ability of ISCs after *Ecc15* infection (Fig. 4C and Supplementary Movie 22). Furthermore, treatment of intestines with TAPI-1, an ADAM and MMP inhibitor^59^, prior to laser ablation, decreased protrusion formation in ISCs (Fig. 4D and Supplementary Movie 23).

**Fig. 5:**
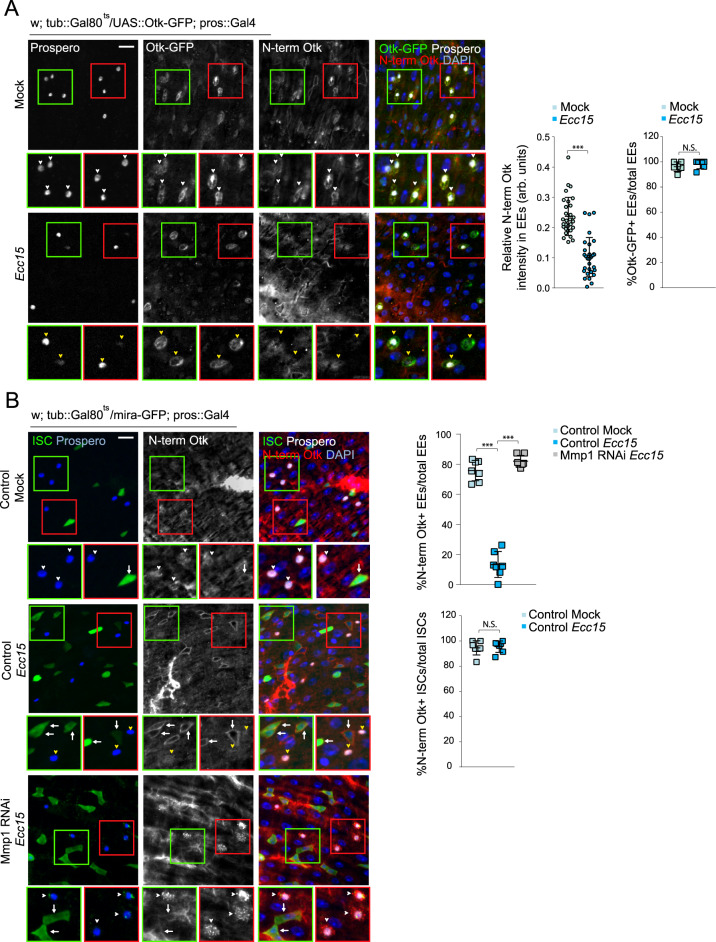
Extracellular Otk is cleaved in EEs by Mmp1 after tissue injury. **A** Full-length Otk with a GFP tag on the C-terminal, intracellular region was overexpressed specifically in EEs to visualize intracellular Otk. Antibodies against the N-terminal, extracellular Otk was used to visualize extracellular Otk. Both N-terminal and C-terminal regions of Otk were detected on the EE cortex in undamaged tissue (white arrowhead), but only C-terminal Otk was detected after tissue damage (yellow arrowhead). **B** N-terminal Otk was detected in EEs from undamaged tissue (white arrowhead), but was largely absent in EEs from damaged tissue (yellow arrowhead). Depleting Mmp1 in EEs from damaged tissue prevented the loss of N-terminal Otk (white arrowhead). N-terminal Otk was detected in ISCs from both undamaged and damaged tissue (white arrows). mean ± SD; *n* = sample size as follows, **A** (Relative N-term Otk intensity): 30 cells from 6 flies from 2 independent experiments, **A** (%Otk-GFP + EEs): 6 flies from 2 independent experiments, **B**: 7 flies from 2 independent experiments; N.S. = not significant (**A** = 0.4043, **B** = 0.7252), ***P < 0.001 (**A**, **B** < 0.0001), based on two-tailed Student’s *t* test (**A**, **B**) and one-way ANOVA with Dunnett test (**B**). Scale bar = 10 µm. Source data are provided as a Source Data file.

To better visualize cleavage of Otk, we overexpressed in EEs full-length Otk tagged with GFP at the intracellular C-terminus and immunostained for the extracellular, N-terminal domain of Otk. Both N-terminal and C-terminal domains were present in EEs in uninjured guts, but N-terminal Otk was no longer present after *Ecc15* infection (Fig. 5A). Crucially, C-terminal Otk was still present, suggesting Otk is still trafficked to the EE membrane (Fig. 5A). The N-terminal domain of endogenous Otk was also detectable in EEs in homeostatic conditions (albeit at a much lower intensity than the overexpressed molecule), but was absent after *Ecc15* infection (Fig. 5B). This loss of N-terminal Otk was no longer observed after EE-specific depletion of Mmp1 (Fig. 5B). Interestingly, in ISCs, the N-terminal domain of Otk was detectable in both uninjured and infected guts, suggesting that Otk is not cleaved in ISCs (Fig. 5B). Overall, these data provide evidence for a crucial role of Mmp1-dependent shedding of Otk in EE cells to activate ISC migration during the regenerative response.

### Impairing ISC migration decreases regeneration rates

**Fig. 6:**
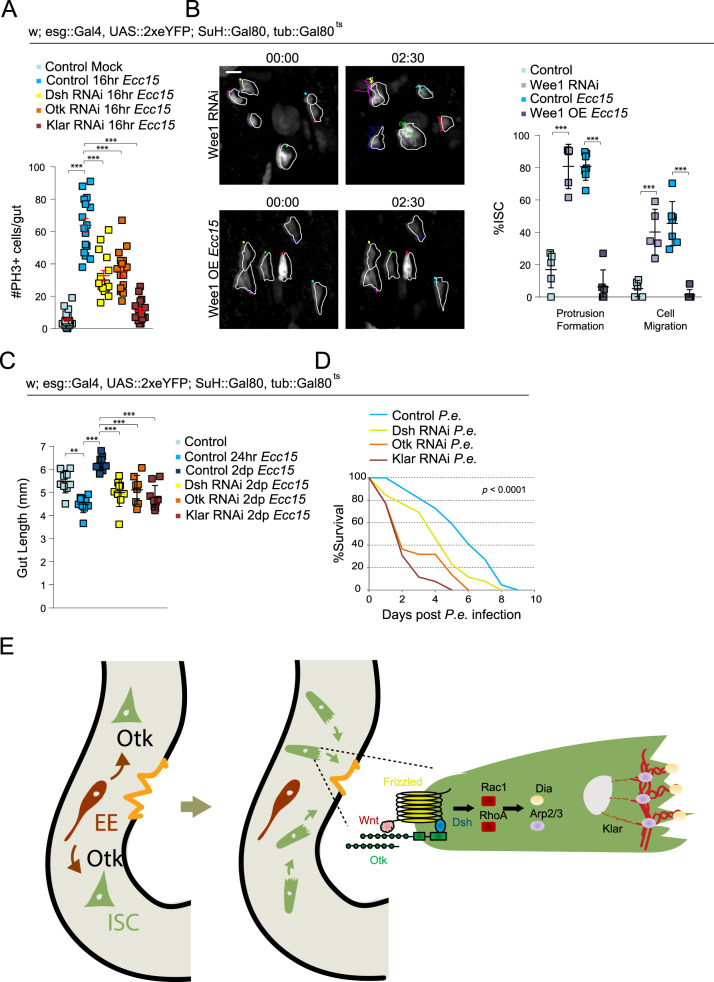
Disrupting ISC migration impairs cell cycle progression and tissue regeneration. **A** Quantification of mitotic numbers after disrupting ISC migration in intestines infected with *Ecc15* for 16hrs. **B** Montage and quantification of ISC migratory behavior after modulating cell cycle progression. OE = overexpression. ISCs outlined in white, and montage tracks measured from the leading edge or, in cells that did not form protrusions, the most distal region of the cell cortex. Mock-treated and *Ecc15*-infected controls were taken from Fig. 1B, as they served as genetic controls. **C** Gut length decreases following 24 hrs of *Ecc15* infection, but overcompensates after 2 days of recovery. Disrupting ISC migration impairs recovery. **D** *P.e*. was fed to 10d females continuously. Disrupting ISC migration decreased survival rates. **E** A model of non-canonical Wnt signaling-mediated regulation of ISC migration following injury. Mmp1 is activated in EE cells, which, in turn, facilitates activation of the non-canonical Wnt pathway, thereby promoting actin rearrangement. mean ± SEM (**A**) or mean ± SD (**B**, **C**); *n* = sample size as follows, **A**: 23 (Control Mock), 16 (Control *Ecc15*, Dsh RNAi, Otk RNAi), and 18 (Klar RNAi) flies from 2 independent experiments, **B**: 5 (Control, Wee1 RNAi), 7 (Control *Ecc15*), and 6 (Wee1 OE) flies from 5 (Control, Control *Ecc15*), 2 (Wee1 RNAi), and 4 (Wee1 OE) independent experiments, **C**: 9 (Control, Control 24 hr, Dsh RNAi, Klar RNAi), 10 (Control 2dp), and 8 (Otk RNAi) flies from 2 independent experiments, D: 44 (Control, Dsh RNAi) and 52 (Otk RNAi, Klar RNAi) flies from a single experiment, repeated two independent times with similar trends. **P < 0.01 (**C**: Control vs Control 24hr = 0.0014), ***P < 0.001 (**A**, **B** – Protrusion Formation, **B** – cell migration: control *Ecc15* vs Wee1 OE, **C**: all except control 2dp vs Otk RNAi < 0.0001, **B** – Cell Migration: Control vs Wee1 RNAi = 0.0008, C: Control 2dp vs Otk RNAi = .0001), based on one-way ANOVA with Dunnett test (**A**), two-tailed Student’s *t* test (**B**), and one-way ANOVA with Tukey test **(C**). Individual *P* values of Fig. 6D = 0.0003 (Control vs Dsh RNAi), < 0.0001 (Control vs Otk RNAi, Control vs Klar RNAi), based on log-rank test (**D**). Scale bar = 10 µm. Timestamp indicated as hours:minutes. Source data are provided as a Source Data file. See also Supplementary Fig. 7, Supplementary Movies 24–25.

To determine whether migration is indeed part of the regenerative response, we tested whether impairing ISC migration would affect tissue regeneration. We first assessed whether the migration of ISCs was coupled to their proliferative activity. Depleting Dsh or Otk decreased the number of mitotic cells after 16 h of *Ecc15* infection (Fig. 6A). Because the non-canonical Wnt pathway plays multiple signaling roles, we also depleted Klar to more specifically target the mechanics of cell migration. Similar to Dsh and Otk, depleting Klar reduced the number of mitotic figures after infection, suggesting a coupling between cell cycle activation and cell migration (Fig. 6A). Proliferative activity is normally very low under homeostatic conditions and depleting Dsh, Otk, or Klar did not significantly decrease phospho-histone H3 (PH3) numbers in uninjured guts (Supplementary Fig. 7a). As an additional assay to test the effect of reduced ISC migration on cell cycle progression, we used an esg-FlipOut lineage tracing system to generate clones^60^. Indeed, disrupting ISC migration by Dsh, Otk, or Klar RNAi reduced the number of cells per clone after *Ecc15* infection (Supplementary Fig. 7b).

To further examine the relationship between cell migration and cell proliferation, we tested whether influencing cell cycle progression directly could also affect ISC migration. Indeed, overexpressing Wee1, a negative regulator of Cdk1^61^, impairs ISC migration after *Ecc15* infection (Fig. 6B and Supplementary Movie 24). Conversely, accelerating G2 progression by depleting Wee1 was sufficient to induce ISC migration in undamaged tissue (Fig. 6B and Supplementary Movie 25). This interdependency of cell cycle progression and cell migration in ISCs indicated that the induction of ISC migration is an integral part of the regenerative response of the intestinal epithelium. To further test this idea, we fed flies with *Ecc15* for 24 h and then transferred them to normal food for 2 days to clear bacteria from the gut. The gut epithelium normally regenerates during this recovery phase, preserving gut size (Fig. 6C and Supplementary Fig. 7c). Depleting Dsh, Otk, or Klar did not affect gut size under homeostatic conditions (Supplementary Fig. 7d), but resulted in a reduced gut size after 2d recovery from *Ecc15* infection (Fig. 6C and Supplementary Fig. 7c). Impairing ISC migration still resulted in impaired regeneration after 1d recovery, but the effect was not as severe as the gut size was still comparable between controls after *Ecc15* infection and 1d recovery (Supplementary Fig. 7d). We then investigated whether this impairment of regeneration also decreased survival rates after infection. Because *Ecc15* is not lethal to flies, we infected animals with *Pseudomonas Entomophila* (*P.e*.), an enteropathogen that induces tissue damage and causes death^62^, for 16 h. Similar to *Ecc15*, *P.e*., induces migration (Supplementary Fig. 7e), and the depletion of Dsh, Otk, or Klar decreased survival rates after *P.e*. infection (Fig. 6D).

## Discussion

Our results uncover a mechanism by which EEs stimulate ISC migration towards an injured area in the intestinal epithelium (Fig. 6E). Injury induces Mmp expression in neighboring EEs, resulting in cleavage and release of the N-terminal domain of Otk. This, in turn, induces protrusion formation and migration in surrounding ISCs by activating Fz2/Dsh signaling, which promotes Rho/Rac-induced lamellipodia formation and Klar-mediated nuclear migration. We propose that the ensuing attraction of ISCs to the site of damage positions them to regenerate missing enterocytes in situ, and our data suggest that this is a critical component of a successful regenerative response in the intestinal epithelium. We further find that ISC migration is coupled to cell cycle progression in ISCs, indicating that the spatial reorganization of cells in the damaged epithelium may also be a prerequisite to the generation of new cells for epithelial replacement.

Our study employs two experimental paradigms to probe ISC migration after epithelial injury: oral enteropathogen infection and localized damage using laser ablation. The epithelial injury induced by *Ecc15* infection induces a robust migratory response by ISCs, but lacks regional specificity due to the high titers of bacteria used in a laboratory setting. This is likely different in the wild, where lower titers of enteropathogens ingested with the food are likely to cause more localized damage. In our study, we sought to mimic such localized damage using laser ablation, which induced migration of ISCs in close vicinity to the injury only but allowed a detailed characterization of directionality of protrusion formation and migration. A caveat of this approach is that the cultured and laser-injured intestines started to deteriorate after 2.5 h of imaging, limiting the ability to track individual ISCs over time. To measure the directionality of movement, we, therefore, had to rely on snapshots of the gut immediately after injury and 4.5 h after ablation, limiting phototoxicity. These experiments revealed that ISCs extended protrusions towards the wound, and migrated towards and accumulated around the wound. However, they could not entirely rule out the possibility that the direction of ISC movement may be stochastic, and that ISCs pause or stop in the vicinity of the wound, resulting in the observed accumulation of ISCs around the wound.

Our data suggest that Wg secreted from EBs and Mmp-mediated release of N-terminal Otk from EEs are critical signals to initiate migration of ISCs after injury, and these secreted molecules may also serve as cues for chemotaxis. Metalloproteases play important roles during cell migration and chemotaxis^63,64^, and increased Mmp1 activity in EEs after injury may also facilitate the release of other signaling molecules. Consistent with a critical role for EE-derived signals, EEs have previously been reported to play important roles in regulating ISC proliferation^53^. Future studies are needed to identify possible additional chemoattractants, as well as to explore other downstream transduction pathways in migrating ISCs.

The exact molecular mechanism by which EE-derived N-terminal Otk induces ISC migration, however, remains unclear. Otk has been reported to be able to homodimerize^33^, and EE-derived Otk may interact with full-length Otk expressed in ISCs to stimulate non-canonical Wnt signaling. As proposed in other settings, Otk may function as co-receptor of Fz for Wnt ligands, stimulating non-canonical Wnt signaling and potentially inhibiting canonical Wnt signaling^27,33^. Importantly, our data suggest that activation of Dsh-mediated Wnt signaling is not required in EEs for the induction of ISC migration, and that the induction of Mmp and the cleavage of Otk are events occurring only in EEs, supporting a model in which EEs sense epithelial injury through an as yet to be identified mechanism, and then attract ISCs to the site of injury through secretion of N-terminal Otk.

In this model, EEs coordinate the directionality of ISC migration, while EBs support migration by serving as a major source of Wnt ligand. EB-derived Wg has been previously reported to be essential for ISC proliferation during regeneration^46^, and our data suggest that it is also required for effective ISC migration. It remains unclear, however, whether EBs serves as the only source of Wg for this process. Both Wnt2 and Wnt4 have been reported to interact with Otk in vitro^27,33^, and these other members of the Wnt family may also be secreted within the *Drosophila* intestine by other cell types to activate non-canonical Wnt signaling in ISCs.

We find that ISC migration and proliferation are closely linked, and that modulating one of these processes affects the other. Because stem cells must divide at the right time and location to ensure proper regeneration, the coordination of migration and division may prevent ISCs from dividing prematurely before migrating closer to the wound. Migration serves to properly space cells within a tissue, and the increased ISC migration after induction of cell cycle progression may also ensure that newly formed cells are properly dispersed in the epithelium. The timing and regulation of this coordination, i.e., whether spatial cues trigger division when cells have reached their destination or whether the cytoskeletal rearrangements triggered during migration invariably induce cell cycle progression, will be fascinating to explore in future studies. Such coordination has been observed during development and in cancer cells through regulation of adhesion signaling by cell cycle-dependent kinases^65–67^. Exploring the mechanisms coordinating the cell cycle and migration in adult stem cells further will deepen our understanding of the regenerative process.

Our data reveal that stem cell migration plays a critical role in the effective regeneration of the adult *Drosophila* intestine. It will be interesting to examine the extent of stem cell migration in adult mammalian tissue, and to determine whether the regulatory mechanisms observed in flies are conserved in these tissues. The migration of stem cells in adult mammalian tissue has largely been centered on hair follicle regeneration of the skin due to its accessibility. While canonical Wnt signaling is required for de novo hair follicle regeneration after wounding, the role of non-canonical Wnt signaling in the wound-induced migration of SCs remains to be characterized^1–6^. Differentiated epithelial cells within the mammalian intestine have recently been reported to actively migrate from the crypt to the top of the villus during steady-state tissue turnover^68^, but whether and how non-canonical Wnt signaling mediates cell migration in the mammalian intestine remains to be established. A role for Wnt5a (a ligand triggering non-canonical Wnt signaling) has been reported to promote homeostasis of the injured colonic epithelium, but it remains unclear whether this phenotype is associated with cell motility^69^. However, the organization of mammalian intestines into specialized regions, with ISCs sequestered at the base of crypts, makes the tissue a poor analog to study the migration of stem cells. In contrast, other barrier tissues, such as the airway epithelium, share similar tissue organization and composition with the fly intestine. The basal stem cells in the mouse trachea, in particular, exhibit many conserved regulatory mechanisms with fly^70^. Exploring the conservation of role and regulation of stem cell migration in these mammalian tissues will improve our understanding of the regenerative response, and will likely lead to the identification of novel targets and strategies for intervention for a wide range of human degenerative diseases.

## Methods

### Lead contact

Further information and requests for resources and reagents should be directed to and will be fulfilled by the Lead Contact, Heinrich Jasper (jasperh@gene.com). Unique biological materials generated in this study are readily available upon request but will require an MTA.

### Experimental model and subject details

#### *Drosophila* stocks, culture, and husbandry

The following lines were obtained from the Bloomington *Drosophila* Stock Center: *UAS::nls-mCherry* (38425), *UAS::mCherry*^*RNAi*^ (35785), *UAS::GFP* (5431), *UAS::CD8-RFP* (27391), *UAS::dsh*^*RNAi*^ (31306), *UAS::eb1-GFP* (35512), *UAS::LifeAct-GFP* (35544), *how::gal4 (*1767)*, UAS::klar*^*RNAi*^ (36721)^71^, *UAS::klar*^*RNAi*^ (28313), *UAS::otk*^*RNAi*^ (25790), *UAS::otk2*^*RNAi*^ (57040), *UAS:: dsh*^*myc*^ (9453), *dsh*^*myc*^ (25385), *UAS::timp* (58708), *UAS::mmp1*^*RNAi*^ (31489)^72^, mmp1^*k04809*^*-lacZ* (12205), *UAS::wee1*^*RNAi*^ (55140)^73^, and *UAS::wee1-VFP* (65390). The following lines were obtained from the Vienna *Drosophila* Stock Center: *UAS::dia*^*RNAi*^ (v20518)^74^, *UAS::arp3*^*RNAi*^ (v108951)^75^, *UAS::rho1*^*RNAi*^ (v12734)^74^, *UAS::rac1*^*RNAi*^ (v49246)^76^, *UAS::dsh*^*RNAi*^ (v101525)^77^, *UAS::otk*^*RNAi*^ (v30833), *UAS::otk2*^*RNAi*^ (v106266), *UAS::fz*^*RNAi*^ (v43075)^78^, *UAS::fz2*^*RNAi*^ (v108988)^79^, *UAS::wg*^*RNAi*^ (v13352)^80^, *UAS::pan*^*RNAi*^ (v3014), and *UAS::arm*^*RNAi*^ (v7767)^81^. The following lines were gifts: *esg::gal4, UAS::2xeYFP; Su(H)::Gal80, tub::Gal80*^*ts*^^82^, *mira-GFP* (Dr. François Schweisguth)^54^, *UAS::otk-GFP* (Dr. Andreas Wodarz)^33^, *tub::Gal80;*^*ts*^ *pros::Gal4* (Dr. Jerome Korzelius)^83^, *mex1-gal4* (Dr. Lucy O’Brien)^15^, *esg::FlipOut*^60^, and *Su(H)GBE::Gal4*^84^. The genotype of flies used in each figure is detailed in Supplementary Data 1. All RNAi lines except for Dsh (31306), Klar (28313), Otk, and Otk2 were previously validated, as referenced, by either qPCR or immunofluorescence. Two different RNAi lines were used for Dsh, Otk, Otk2, and Klar to minimize off-target effects. Lines 25790, 28313, and 57040 were only used for Supplementary Fig. 4b. Line 31306 was used for Fig. 3c, Supplementary Fig. 4b, and Supplementary 5d.

Flies were raised at 18 °C to prevent RNAi or protein expression during development and 65% humidity, on a 12-hour light/dark cycle. To induce genetic expression, flies were shifted to 29 °C for 3 days prior to bacterial infection or mock-treatment (for a total of ~4 days induction). Otherwise, flies were shifted from 18 °C to 29 °C for 4 days. Standard fly food was prepared with the following recipe: 1 L distilled water, 7.3 ml EtOH, 13 g agar, 22 g molasses, 65 g malt extract, 18 g brewer’s yeast, 80 g cornflour, 10 g soy flour, 6.2 ml propionic acid, and 2 g methyl-*p*-benzoate. Flies were manipulated by CO_2_ anesthesia. Only female flies were used in this study because females exhibit higher intestinal turnover rates than males. All flies were dissected at 10d except for gut size experiments, which were dissected at 11d.

### Method details

#### Drosophila Ecc15 and P.e. infection

For *Ecc15* infection, *Ecc15* was cultured in 15 ml of LB medium for 16 h at 30°. The culture was pelleted, resuspended in 400 µL of 5% sucrose in water, and fed to two-hour starved flies on Whatman paper for 16 h. Intestines were subsequently dissected. For gut size experiments, flies were fed with *Ecc15* for 24 h before being transferred to standard fly food for up to 48 h. For *P.e*. infection, *P.e*. was cultured in 20 ml of LB medium for 16 h at 30°. The culture was pelleted, resuspended in 400 µL of 5% sucrose in water, and fed to two-hour starved flies for either 16 h (for live imaging experiments) or the duration of the experiment (for survival experiments). For survival experiments, 40–60 10d flies were housed together and continuously fed with *P.e*, with 300 µl of 5% sucrose in water added daily. Dead flies were counted daily. For mock controls, flies were starved for two hours and fed 5% sucrose on a vial containing Whatman filter paper for 16 h. Survival experiments were repeated three times.

#### Live imaging of *Drosophila* intestines

Intestines from adult female flies were dissected in culture media containing 2 mM CaCl_2_, 5 mM KCl, 5 mM HEPES, 8.2 mM MgCl_2_, 108 mM NaCl, 4 mM NaHCO_3_, NaH_2_PO_4_, 10 mM sucrose, 5 mM trehalose, and 2% fetal bovine serum (Adult Hemolymph-like Saline, AHLS). Intestines were transferred to a 35 mm glass-bottom dish (MatTek, P35G-1.5-14-C), embedded in 3% low melting agarose (in AHLS), and submerged in AHLS. The posterior midgut was imaged at intervals of 10 min for up to three hours on a Yokogawa CSU-W1/Zeiss 3i Marianas spinning disk confocal microscopy system with a 40x PlanFleur objective or an Andor CSU-X1/Nikon Ti-E spinning disk confocal microscopy system with a 40x PlanFleur objective, or, for laser ablation experiments, on a Bruker Ultima In Vivo Multiphoton/Newport MaiTai DeepSee Spectra Physics two-photon microscopy system with a 20x Olympus 1.0 water immersion objective.

#### Laser ablation of *Drosophila* intestines

Ablated intestines and corresponding undamaged controls were imaged with a 960 nm wavelength, 2.8 µs dwell time, and ~25 mW power, using a Bruker Ultima In Vivo Multiphoton/Newport MaiTai DeepSee Spectra Physics 2-photon microscopy system with a 20x Olympus 1.0 water immersion objective. A wound of a ~30 µm diameter was created in the posterior midgut with an 880 nm wavelength, 20 µs dwell time, ~278 mW power, and 60× zoom on the same system. Ablated guts were live imaged on the two-photon system at intervals of 10 min for ~2.5hrs. For fixed analysis, ablated guts were cultured in AHLS for either 1 hr or 4.5 hrs prior to fixation.

#### Treatment of *Drosophila* intestine with small molecule inhibitors and recombinant protein

Intestines from adult female flies were dissected in AHLS (see Live Imaging of *Drosophila* intestines) and treated with small molecule inhibitors or recombinant protein in AHLS 30 min prior to imaging and throughout the live imaging process. Mock-treated intestines were incubated in 0.1% dimethylsulfoxide (DMSO) in AHLS 30 min prior to imaging and throughout the live imaging process.

Small molecule inhibitors and concentrations used in this study: Blebbistatin (20 µM, Sigma Aldrich, B0560), Cytochalasin B (10 µM, Sigma Aldrich, C6762), LGK974 (1 µM, Millipore Sigma Chemicals, 531091), and TAPI-1 (20 µM, EMD Millipore, 59053). Recombinant protein and concentrations used in this study: C-terminal Ptk7 (1 µg/ml, see Recombinant C-terminal Ptk7 generation).

#### Immunostaining of *Drosophila* intestine

Intestines from adult female flies were dissected in PBS (Phosphate-buffered saline, pH 7.4), and fixed for 25 min at room temperature in media containing 100 mM glutamic acid, 25 mM KCl, 20 mM MgSO_4_, 4 mM sodium phosphate, 1 mM MgCl_2_, and 4% formaldehyde. Intestines were washed with PBS and stained in PBS, 0.3% Triton X100 supplemented with 5% donkey serum. Intestines were incubated in primary antibody overnight at 4 °C antibodies, washed in PBS + 0.01% Tween-20, and incubated in secondary antibodies and Hoechst stain (1:1000) for 2 h at room temperature.

Antibodies used in this study: Mouse monoclonal against Prospero (1:300, DHSB, MR1A, RRID:AB_528440), Myc (1:300, Abcam, ab32, RRID:AB_303599), and Mmp1 (1:10, DHSB, 5H7B11, RRID: AB_579779; 3A6B4, RRID:AB_579780; 3B8D12, RRID: AB_579781); rabbit polyclonal against ß-galactosidase (1:300, MP Biomedicals, 085597-CF), phospho-histone H3 (1:1000, EMD Millipore, 06–570, RRID:AB_310177), and Otk (1:50, see Otk antibody generation); and chicken polyclonal against GFP (1:300, Abcam, ab13970, RRID:AB_300798). Secondary antibodies were cyanine dyes from Jackson ImmunoResearch Laboratories (1:300). Anti-GFP antibodies were used to amplify the signal in cells overexpressing GFP-tagged Otk. For Mmp1 staining, all three Mmp1 antibodies were used in conjunction at 1:10 in a 1:1:1 mixture as previously reported^85^.

#### Otk antibody and recombinant Ptk7 generation

To generate polyclonal Otk antibodies, rabbits were immunized against immunogens consisting of amino acids 23–581 for recognition of the extracellular domain or 632–1033 for recognition of the intracellular domain (Genentech, Inc. Protein Sciences). Sera were extracted and purified using Protein A, resulting in IgG polysera. Reactivity was confirmed by immunofluorescence. To generate recombinant murine extracellular Ptk7, Met1-Glu683 was cloned into a modified pRK vector containing a CMV promoter and C-terminal Flag tag (Genentech, Inc. Protein Sciences). Protein was expressed in HEK293 cells using standard transfection protocols and the supernatant was harvested after seven days. Clarified supernatant was passed over Flag-resin equilibrated with buffer A (25 mM Tris pH 7.5, 150 mM NaCl, 5 mM EDTA), washed with buffer B (25 mM Tris pH 7.5, 150 mM NaCl, 5 mM EDTA, 0.2% Triton X-114, 0.2% Triton X100), eluted in buffer C (50 mM sodium citrate, 150 mM NaCl, pH 3.0), and neutralized by addition of 1 M Tris pH 8.0. Protein was concentrated and passed over a Superdex 200 column in phosphate-buffered saline, and the peak fraction was collected. Sodium dodecyl sulphate–polyacrylamide gel electrophoresis and mass spectrometry (ISD MALDI) were used to confirm the high purify and identity of the protein (starting at residue Ala23).

#### Esg::FlipOut induction

Flies were raised at 18 °C and shifted to 29 °C for three days starting at 6d. Flies were then fed *Ecc15* in 5% sucrose or 5% sucrose alone at 9d and maintained at 29 °C for 24 hrs before dissecting. The number of cells per clone was quantified.

#### Microscopy and image analysis

Images of fixed tissue were taken either with a Yokogawa CSU-W1/Zeiss 3i Marianas spinning disk confocal microscopy system or a Andor CSU-X1/Nikon Ti-E spinning disk confocal microscopy system. Intestines were imaged using a ×40 PlanFleur objective or, for gut size measurements, ×10 PlanFleur objective. Images were analyzed and processed using ImageJ (NIH, Bethesda, MD) and Adobe Photoshop. Figures were composed in Adobe Illustrator. Except for quantification of PH3+ cells and gut length, which was performed in the entire gut, only the R4 region of the posterior midgut was analyzed for consistency.

### Quantification and statistical analysis

All quantifications were performed manually using ImageJ software (NIH, Bethesda, MD). Except for quantification of PH3+ cells and gut length, which was performed in the entire gut, only the R4 region of the posterior midgut was analyzed for consistency.

#### Quantification of gut size and immunostaining

Intestines were dissected from females at 10d of age, except for gut length analyses, which were at 11d. Mitotic numbers were counted from the entire gut as determined by phospho-histone H3 positivity. The gut length was measured from the beginning of the anterior midgut to the end of the posterior midgut. The presence of cortical Dsh was identified by localization of Dsh around the periphery of the ISC, as determined by cytoplasmic GFP expression. Cell-type-specific expression of Otk and Mmp1 was determined by ISC- and EE-specific expression of GFP, or prospero immunostaining for EEs. Dsh and Mmp1 expression in ablated intestines was only quantified within a 100 µm radius of the ablation site. For measurement of N-terminal Otk and Mmp1 intensity, fluorescence levels were set to the same baseline across all images. Relative N-terminal Otk levels were measured by normalizing the corrected total cell fluorescence (CTCF) of N-terminal Otk in the cell outline with the CTCF of C-terminally GFP-tagged Otk. Relative Mmp1 levels were measured by normalizing the mean fluorescence of Mmp1 in the cell outline with the mean fluorescence of the same area in adjacent regions.

#### Analysis of intestinal stem cell migration

Only the R4 region of the posterior midgut, chosen randomly, was imaged. Quantification of migratory behavior consisted of assaying for protrusion formation and cell migration and was only performed on guts with minimal sample movement as determined by autofluorescence of the tissue. ISCs were scored as exhibiting protrusion formation if a protrusion was extended at any point during a 2.5 hr live imaging duration. ISCs were scored as exhibiting cell migration if the cell body translocated at least 3 µm during a 2.5 hr live imaging duration, using the tissue background as a reference point, if necessary. Tracks were measured every ten minutes using the Manual Tracking plugin (Fabrice Cordeli, Curie Institute, Paris, France) in ImageJ (NIH, Bethesda, MD), tracing either the edge of the protrusion or the center of the cell body. The starting point of each ISC was designated as the center of the Cartesian plane (0,0), and the x,y coordinate of the ISC at each time point was normalized to its starting point. For ISCs that did not exhibit protrusion formation, the most distal region of the cell cortex was tracked instead. For laser ablation experiments, ISC migratory behavior was assayed within 75 µms from the site of injury.

#### Analysis of migration directionality

To assay ISC accumulation around the periphery of the wound in fixed, ablated intestines (Supplementary 2c), the ISC numbers within a 40 µm radius of the ablation site were counted and compared with the ISC numbers within the same area of the contralateral side of the intestine. ISCs within the ablation site area on the contralateral side were not counted. For undamaged controls, ISCs were counted on contralateral sides of the intestine in a 200 µm × 200 µm area. To quantify the angle of ISC protrusions in relation to the wound of an ablated gut (Supplementary Fig 2a), the angle was measured between the vector of the shortest distance from the center of the cell body to the periphery of the wound and the vector from the center of the cell body to the center of the leading edge. To plot ISC position in ablated tissue (Supplementary 2b), the periphery of the wound closest to a given ISC was designated as the center of the Cartesian plane (0,0). The starting position of the ISC was the x,y coordinate of the cell cortex closest to the wound immediately after ablation, and normalized to the x,y coordinate of the wound periphery closest to the ISC. The ending position of the ISC was the x,y coordinate of the cell cortex closest to the wound 4.5 hrs post-ablation, and normalized to the x,y coordinate of the wound periphery closest to the ISC. For example, if the x,y coordinate of an ISC was measured at 108, 148 and the wound was measured at 133, 148, the wound would be set at 0,0 and the normalized x,y coordinate of the ISC would be −25, 0. In control, unablated tissue, a single arbitrary point in the middle of the gut was designated as the center of the Cartesian plane (0,0). ISC position was determined by the x,y coordinate of the cell cortex closest to the designated point, and normalized to the x,y coordinate of the designated point. Change in displacement was calculated from the difference in distance between the starting ISC position and the Cartesian origin, and the ending ISC position and the Cartesian origin.

#### Statistical analysis and reproducibility

Each sample, “*n*”, is from at least two independent experiments, and is defined in the Figure and Supplementary Figure Legends depending on the experiment. All sets of experimental repeats yielded similar results. Additional statistical details for each experiment are also noted in the Figure and Supplementary Figure Legends. Statistical analyses were performed with Prism (GraphPad Software, La Jolla, CA, USA). A two-tailed Student’s *t* test was used to determine statistical significance between two independent groups. A one-way ANOVA with a Tukey or Dunnett test was used to determine statistical significance with multiple comparisons between three or more independent groups. A chi-square test was used to test for statistical significance when comparing data according to a set hypothesis (percentage of protrusions formed towards versus away from wound). A log-rank test was used to test for statistical significance in *P.e*. survival assays. Significance was accepted at the level of *p* < 0.05. No statistical methods were used to predetermine sample sizes, but our sample sizes are similar to those generally employed in the field. The specific region imaged was chosen at random within the R4 of the posterior midgut. All experiments (from both live and fixed samples) were quantified blindly.

### Reporting summary

Further information on research design is available in the Nature Research Reporting Summary linked to this article.

## Supplementary information

Supplementary Information

Description of Additional Supplementary Files

Supplementary Data 1

Supplementary Movie 1

Supplementary Movie 2

Supplementary Movie 3

Supplementary Movie 4

Supplementary Movie 5

Supplementary Movie 6

Supplementary Movie 7

Supplementary Movie 8

Supplementary Movie 9

Supplementary Movie 10

Supplementary Movie 11

Supplementary Movie 12

Supplementary Movie 13

Supplementary Movie 14

Supplementary Movie 15

Supplementary Movie 16

Supplementary Movie 17

Supplementary Movie 18

Supplementary Movie 19

Supplementary Movie 20

Supplementary Movie 21

Supplementary Movie 22

Supplementary Movie 23

Supplementary Movie 24

Supplementary Movie 25

Reporting Summary

## Data Availability

This study did not generate any data sets. Source data are provided with this paper.

## Source data

Source Data

## Code availability

This study did not generate any code.
