# Supplementary material for: Non-canonical Wnt signaling promotes directed migration of intestinal stem cells to sites of injury
Source: Nat Commun. 2021 Dec 9;12:7150. doi: 10.1038/s41467-021-27384-4 (PMC8660829; doi:10.1038/s41467-021-27384-4)
Supplement: Supplementary file 1 — Supplementary Information [file 41467_2021_27384_MOESM1_ESM.pdf]

Supplementary Information for

Non-canonical Wnt signaling promotes directed migration of intestinal stem cells to sites of injury.

Daniel Jun-Kit Hu[1], Jina Yun[1], Justin Elstrott[1], and Heinrich Jasper[1,*,#]

[1]Genentech Inc., 1 DNA Way, South San Francisco, CA 94080
[*]Corresponding Author
[#]Lead Contact
Email: jasperh@gene.com
Phone: (650) 467-1248

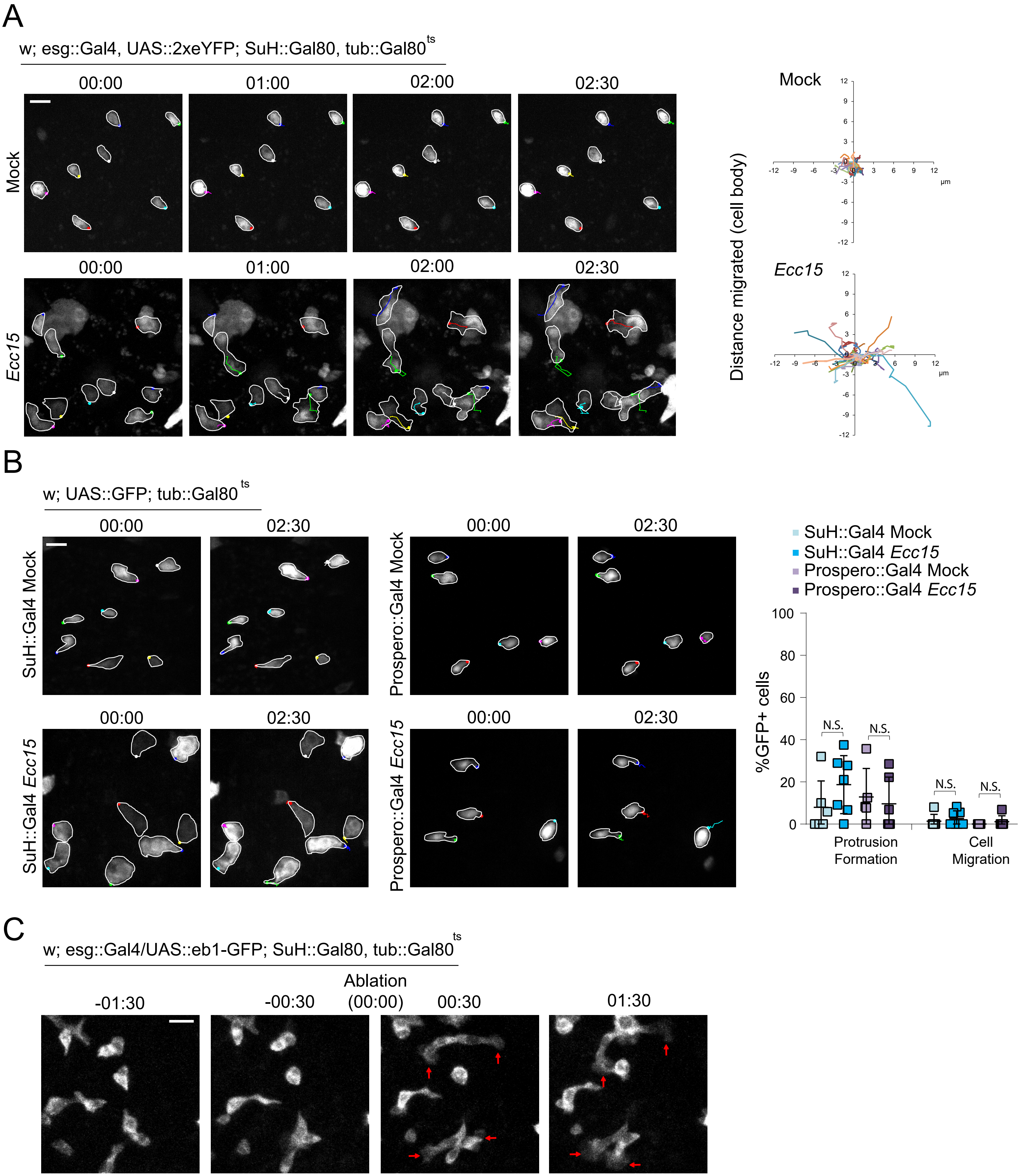
A
w; esg::Gal4, UAS::2xeYFP; SuH::Gal80, tub::Gal80ts
00:00
01:00
02:00
02:30
Mock
Ecc15
Distance migrated (cell body)
µm
B
w; UAS::GFP; tub::Gal80ts
SuH::Gal4 Mock
SuH::Gal4 Ecc15
Prospero::Gal4 Mock
Prospero::Gal4 Ecc15
%GFP+ cells
N.S.
Protrusion Formation
Cell Migration
C
w; esg::Gal4/UAS::eb1-GFP; SuH::Gal80, tub::Gal80ts
-01:30
-00:30
Ablation (00:00)
00:30
01:30

Supplementary Figure 1: Additional characterization of cell migration in *Drosophila* intestine, Related to Figure 1.

A. Montage of ISC migration and tracings of the displacement of the ISC body in mock-treated and *Ecc15*-infected intestines. ISCs outlined in white, and montage tracks measured from the leading edge or, in cells that did not form protrusions, the most distal region of the cell cortex. Starting x,y coordinate of ISCs normalized to 0,0. Data are representative of three independent experiments. B. Montage and quantification of EB and EE migratory behavior in mock-treated and *Ecc15*-infected intestines. GFP+ cells outlined in white, and montage tracks measured from the most distal region of the cell cortex. C. Montage of cytoskeleton dynamics pre- and post-ablation. Ablation occurred at time point 00:00. Red arrows indicate protrusions. Data are representative of three independent experiments. mean ± SD; n=6 (SuH::Gal4 mock, Prospero::Gal4 *Ecc15*), 7 (SuH::Gal4 *Ecc15*), and 5 (Prospero::Gal4 mock) flies from 2 independent experiments; N.S. = not significant (B – Protrusion Formation: SuH::Gal4 mock vs SuH::Gal4 *Ecc15* = 0.1709, B – Protrusion Formation: Prospero::Gal4 mock vs Prospero::Gal4 *Ecc15* = 0.6925, B – Cell Migration: SuH::Gal4 mock vs SuH::Gal4 *Ecc15* = 0.4769, B – Cell Migration: Prospero::Gal4 mock vs Prospero::Gal4 *Ecc15* = 0.3893), based on two-tailed Student's t-test. Scale bar = 10μm. Timestamp indicated as hours:minutes. Source data are provided as a Source Data file.

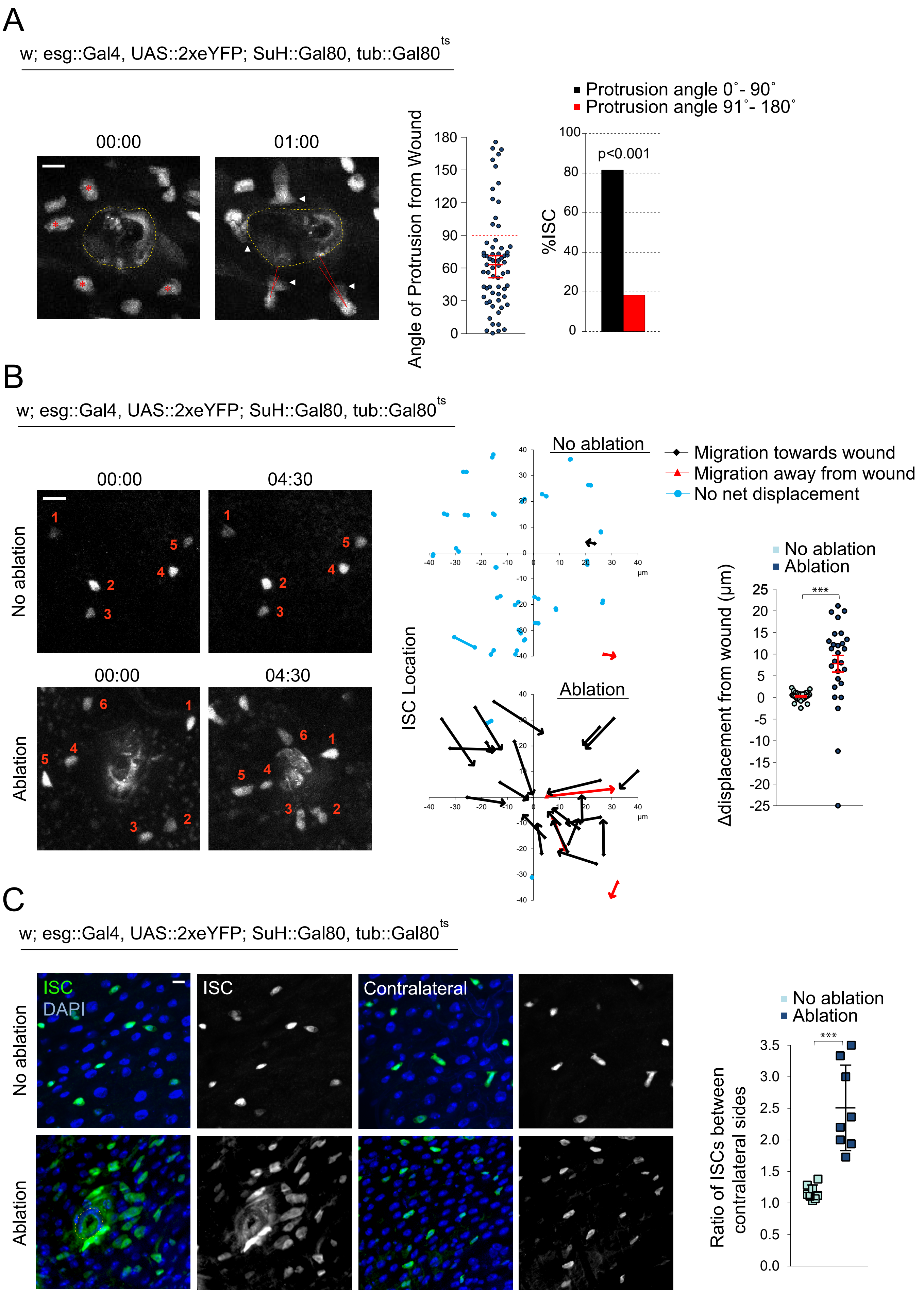
A
w; esg::Gal4, UAS::2xeYFP; SuH::Gal80, tub::Gal80ts
00:00
01:00
Angle of Protrusion from Wound
Protrusion angle 0˚- 90˚
Protrusion angle 91˚- 180˚
p<0.001
%ISC
B
w; esg::Gal4, UAS::2xeYFP; SuH::Gal80, tub::Gal80ts
00:00
04:30
No ablation
Ablation
ISC Location
Migration towards wound
Migration away from wound
No net displacement
Δdisplacement from wound (μm)
***
C
w; esg::Gal4, UAS::2xeYFP; SuH::Gal80, tub::Gal80ts
ISC
DAPI
Contralateral
Ratio of ISCs between contralateral sides

Supplementary Figure 2: Directionality of ISC migration towards sites of injury, Related to Figure 1.

A. Montage and quantification of ISC protrusion angle in relation to the wound in ablated tissue. Red asterisk indicate ISCs that will form protrusions after 1hr. Red line depict angle of protrusion in relation to wound, with 0° indicating a protrusion forming along the shortest distance towards the wound. Angles are measured between the vector of the cell body to the middle of the protrusion with the vector of the cell body to the closest point of the wound periphery. B. Montage and quantification of ISC position and change of displacement from the wound immediately following laser ablation (start point) and 4.5hrs later (end point). Numbers in montage correlate to the corresponding ISC at start and end points. The origin of the arrow in the Cartesian plane indicates the ISC start point and the tip of the arrow indicates the ISC end point. x,y coordinates of ISC position in unablated tissue is normalized to an arbitrary point in the middle of the gut, which is set at 0,0. x,y coordinates of ISC position in ablated tissue is normalized to the closest point to the wound periphery of a given ISC, which is set at 0,0. Delta displacement is the difference in distance between the start point and the Cartesian origin, and the end point and the Cartesian origin. C. ISCs accumulated at the site of injury at 4.5hrs post ablation, with increased ISC number around the wound compared to the same area on the contralateral side. median ± 95% CI (A), mean ± SEM (B), or mean ± SD (C); n = sample size as follows, A: 65 cells from 6 flies from 6 independent experiments, B: 27 cells from 4 (no ablation) or 5 (ablation) flies from two independent experiment, C: 8 flies from 2 (no ablation) and 3 (ablation) independent experiments; The P value of Supplementary Figure 2B < 0.0001 with Chi squared = 23.4 and 1 degree of freedom, based on chi-square test (A), ***P<0.001 (B = 0.0006, C < 0.0001), based on two-tailed Student's t-test (B,C). Scale bar = 10μm. Timestamp indicated as hours:minutes. Source data are provided as a Source Data file.

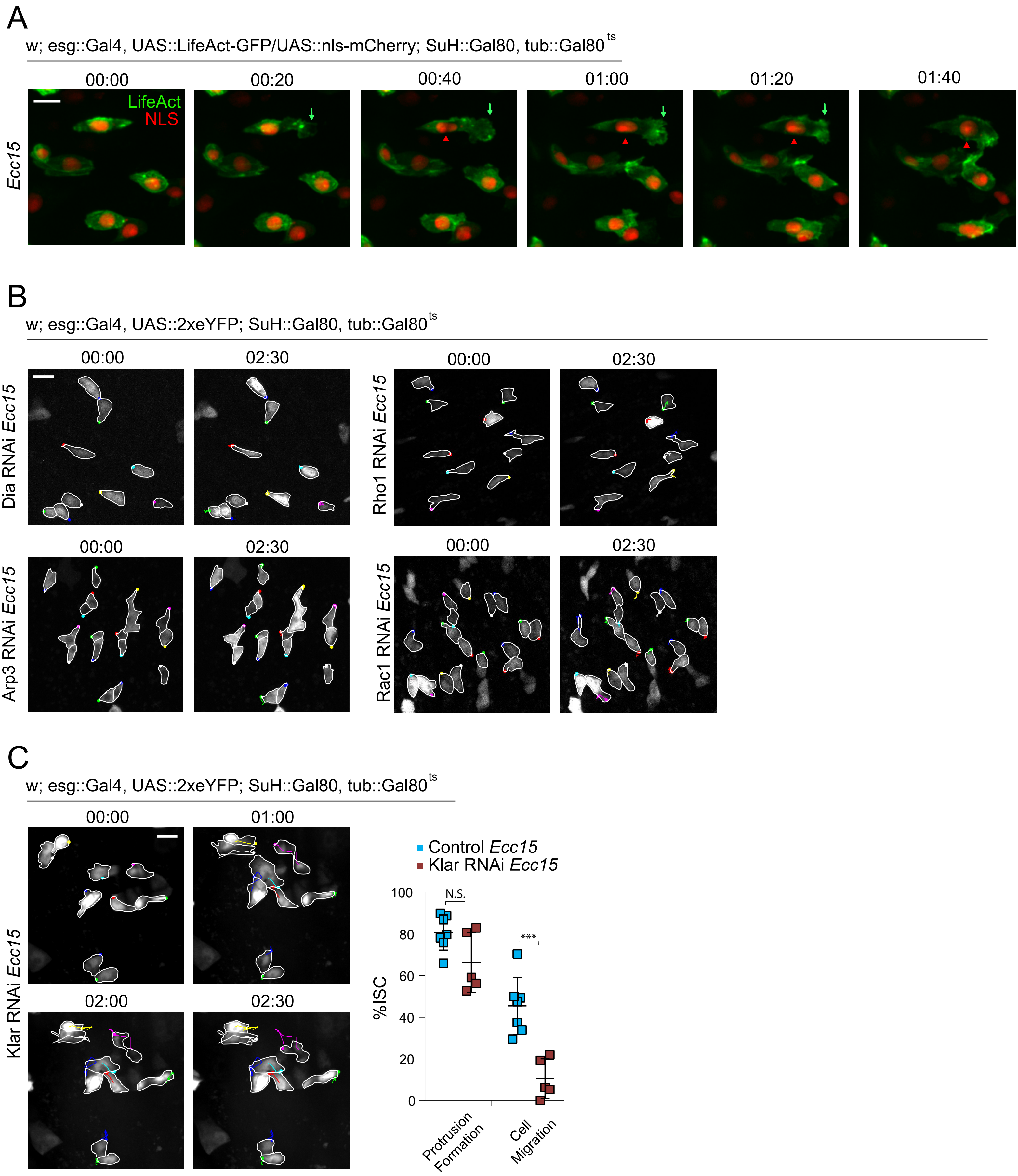

A
w; esg::Gal4, UAS::LifeAct-GFP/UAS::nls-mCherry; SuH::Gal80, tub::Gal80ts
00:00
00:20
00:40
01:00
01:20
01:40
LifeAct
NLS
Ecc15
B
w; esg::Gal4, UAS::2xeYFP; SuH::Gal80, tub::Gal80ts
00:00
02:30
Dia RNAi Ecc15
Rho1 RNAi Ecc15
Arp3 RNAi Ecc15
Rac1 RNAi Ecc15
C
w; esg::Gal4, UAS::2xeYFP; SuH::Gal80, tub::Gal80ts
00:00
01:00
02:00
02:30
Klar RNAi Ecc15
Control Ecc15
Klar RNAi Ecc15
N.S.
***
%ISC
100
80
60
40
20
0
Protrusion Formation
Cell Migration

Supplementary Figure 3: Additional analyses of cytoskeleton-associated proteins during ISC migration, Related to Figure 1.

A. Montage of ISC migration in *Ecc15*-infected intestines. Green arrows depict leading edge, and red arrowheads depict migrating nucleus. Data are representative of two independent experiments. B. Montage of ISC migratory behavior after disrupting actin assembly in *Ecc15*-infected intestines. ISCs outlined in white, and montage tracks measured from the most distal region of the cell cortex. C. Montage and quantification of ISC migration in *Ecc15*-infected Klar^{RNAi} intestines. ISCs outlined in white, and montage tracks measured from the most distal region of the cell cortex. *Ecc15*-infected controls were taken from Figure 1B, as they served as genetic controls and were performed in parallel. mean ± SD; n=7 (Control) and 5 flies (Klar RNAi) from 5 and 3 independent experiments respectively; N.S. = not significant (C – Protrusion Formation = 0.0536), ***P<0.001 (C – Cell Migration = 0.0006), based on two-tailed Student's t-test. Scale bar = 10µm. Timestamp indicated as hours:minutes. Source data are provided as a Source Data file.

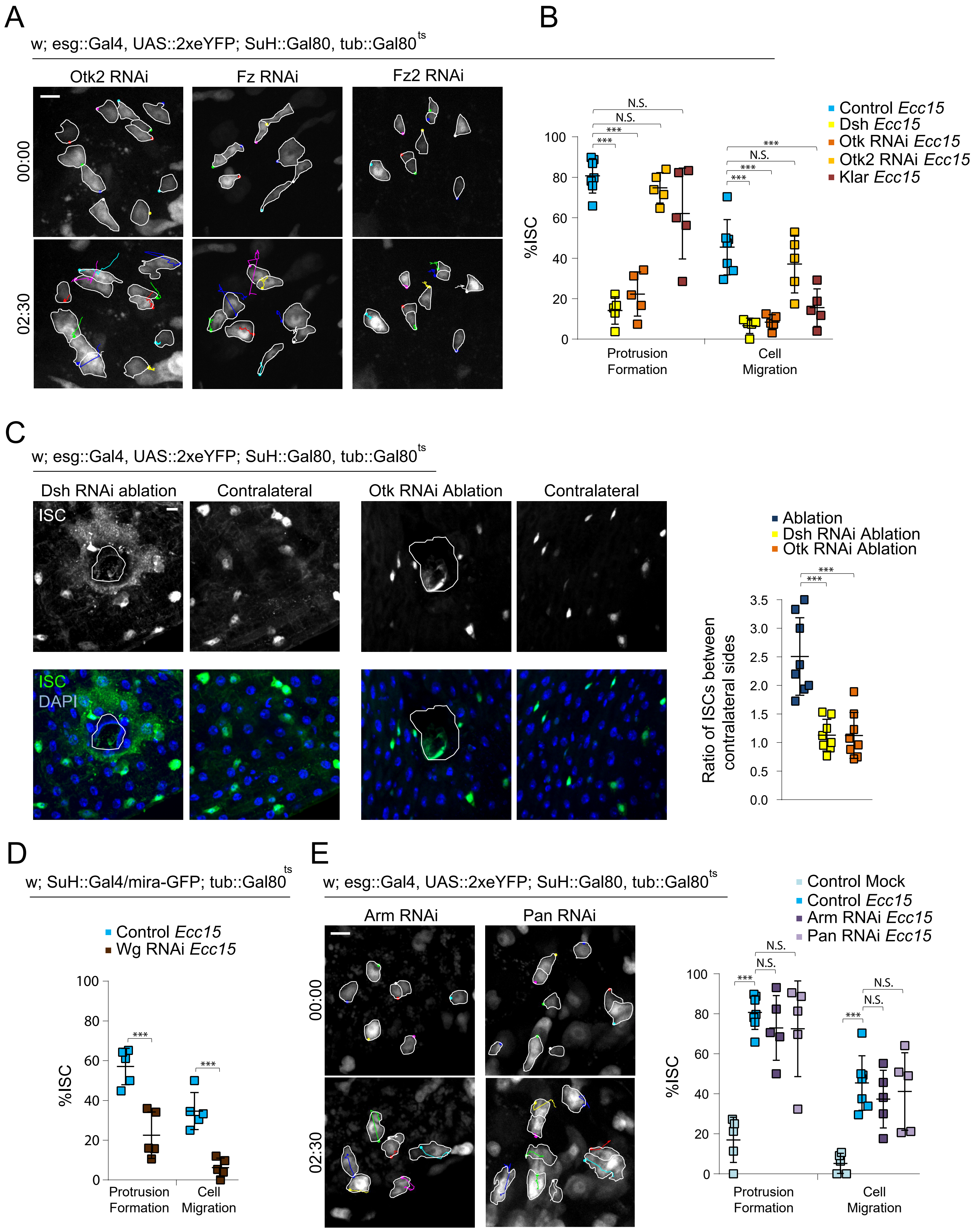
A
w; esg::Gal4, UAS::2xeYFP; SuH::Gal80, tub::Gal80ts
Otk2 RNAi
Fz RNAi
Fz2 RNAi
00:00
02:30
B
Control Ecc15
Dsh Ecc15
Otk RNAi Ecc15
Otk2 RNAi Ecc15
Klar Ecc15
%ISC
N.S.
***
Protrusion Formation
Cell Migration
C
w; esg::Gal4, UAS::2xeYFP; SuH::Gal80, tub::Gal80ts
Dsh RNAi ablation
Contralateral
Otk RNAi Ablation
Contralateral
ISC
DAPI
Ablation
Dsh RNAi Ablation
Otk RNAi Ablation
Ratio of ISCs between contralateral sides
D
w; SuH::Gal4/mira-GFP; tub::Gal80ts
Control Ecc15
Wg RNAi Ecc15
E
w; esg::Gal4, UAS::2xeYFP; SuH::Gal80, tub::Gal80ts
Arm RNAi
Pan RNAi
Control Mock
Control Ecc15
Arm RNAi Ecc15
Pan RNAi Ecc15

Supplementary Figure 4: Additional analyses of Wnt signaling roles during ISC migration, Related to Figure 2.

A. Montage of ISC migration after genetic perturbation in *Ecc15*-infected intestines. ISCs outlined in white, and montage tracks measured from the leading edge or, in cells that did not form protrusions, the most distal region of the cell cortex. B. In *Ecc15*-infected intestines, expression of alternate RNAi against Dsh, Klar, and Otk impaired ISC migration while alternate RNAi against Otk2 had no effect. *Ecc15*-infected controls were taken from Figure 1B, as they served as genetic controls. C. ISCs from Dsh^{RNAi} or Otk^{RNAi} intestines no longer accumulated at the periphery of the wound (outline) 4.5hrs post ablation. Data from ablation control were taken from Supplementary Figure 2C, as they served as genetic controls and were performed in parallel. D. Depleting Wg specifically in EBs impaired ISC migration. E. Montage and quantification of ISC migratory behavior in *Ecc15*-infected flies with disrupted canonical Wnt signaling. ISCs outlined in white, and montage tracks measured from the leading edge or, in cells that did not form protrusions, the most distal region of the cell cortex. E. Montage and quantification of ISC migratory behavior in *Ecc15*-infected flies with disrupted canonical Wnt signaling. ISCs outlined in white, and montage tracks measured from the leading edge. Controls were taken from Figure 1B, as they served as genetic controls. mean ± SD; n= sample size as follows, B: 7 (Control) and 5 (all other conditions) flies from 5 (Control), 3 (Dsh RNAi, Otk2 RNAi, Klar RNAi), and 2 (Otk RNAi) independent experiments, C: 8 flies from 3 (Ablation, Otk RNAi) and 2 (Dsh RNAi) independent experiments, D: 5 flies from 4 independent experiments, E: 5 (All conditions except Control *Ecc15*) and 7 (Control *Ecc15*) flies from 5 (Control Mock, Control *Ecc15*) and 2 independent experiments (Pan RNAi, Arm RNAi). N.S. = not significant (B – Protrusion formation: Control *Ecc15* vs Otk2 RNAi = 0.8285, Control *Ecc15* vs Klar RNAi = 0.0558, B – Cell Migration: Control *Ecc15* vs Otk2 RNAi = 0.4692, E – Protrusion Formation: Control *Ecc15* vs Arm RNAi = 0.7239, Control *Ecc15* vs Pan RNAi = 0.6954, E – Cell Migration: Control *Ecc15* vs Arm RNAi = 0.6439, Control *Ecc15* vs Pan RNAi = 0.9157), ***P<0.001 (B – Protrusion Formation: Control *Ecc15* vs Dsh RNAi, Control *Ecc15* vs Otk RNAi, B – Cell Migration: Control *Ecc15* vs Dsh RNAi, Control *Ecc15* vs Otk RNAi, C,E < 0.0001, B – Cell Migration: Control *Ecc15* vs Klar RNAi = 0.0003, D –

Protrusion Formation = 0.0008, D – Cell Migration = 0.0003), based on one-way ANOVA with Dunnett test (B,C,E) and two-tailed Student's t-test (D). Scale bar = 10µm. Timestamp indicated as hours:minutes. Source data are provided as a Source Data file.

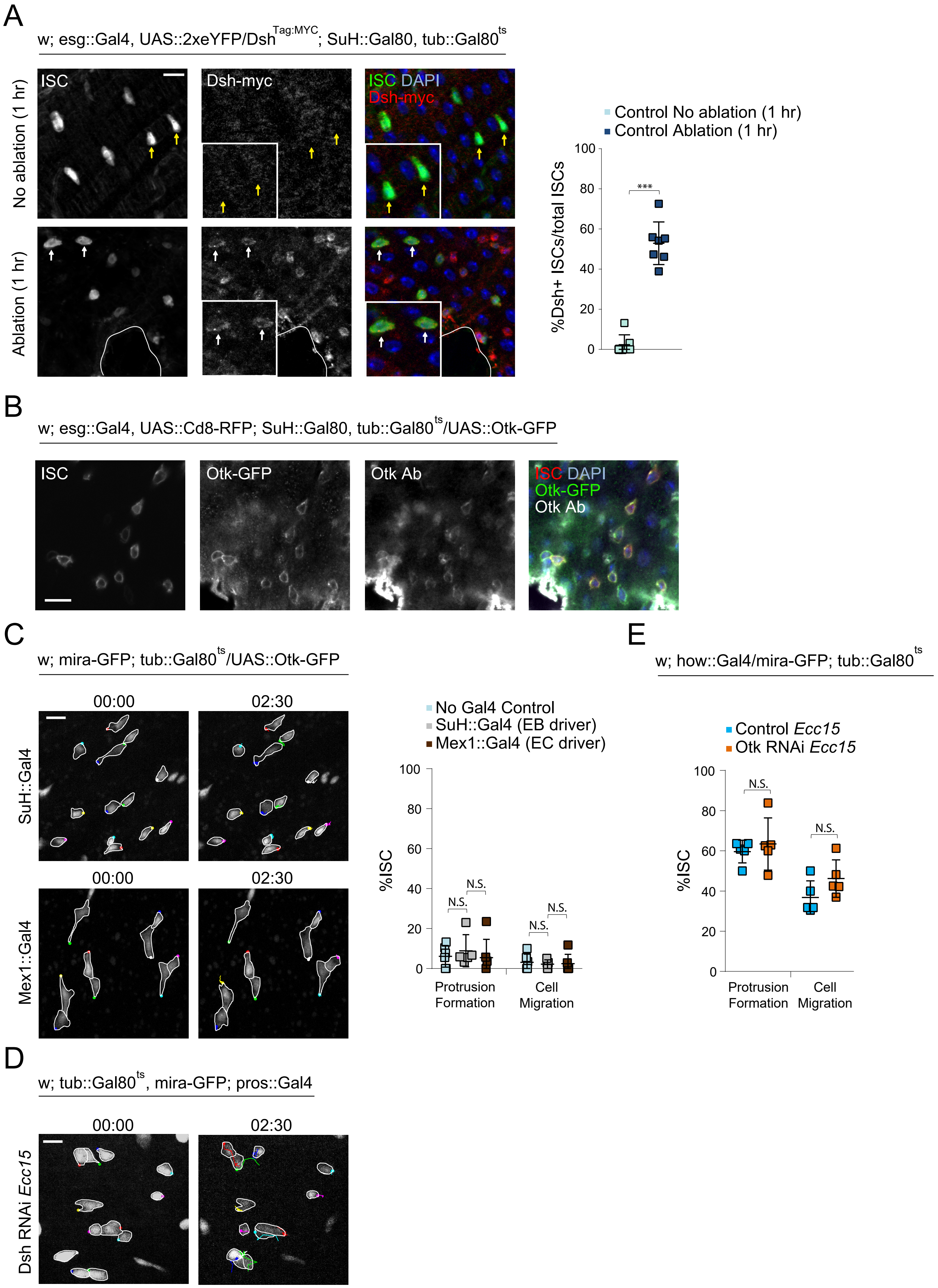
A
w; esg::Gal4, UAS::2xeYFP/Dsh Tag:MYC; SuH::Gal80, tub::Gal80 ts
No ablation (1 hr)
Ablation (1 hr)
ISC
Dsh-myc
ISC DAPI
Dsh-myc
Control No ablation (1 hr)
Control Ablation (1 hr)
%Dsh+ ISCs/total ISCs
***
100
80
60
40
20
0
B
w; esg::Gal4, UAS::Cd8-RFP; SuH::Gal80, tub::Gal80 ts/UAS::Otk-GFP
ISC
Otk-GFP
Otk Ab
ISC DAPI
Otk-GFP
Otk Ab
C
w; mira-GFP; tub::Gal80 ts/UAS::Otk-GFP
00:00
02:30
SuH::Gal4
Mex1::Gal4
No Gal4 Control
SuH::Gal4 (EB driver)
Mex1::Gal4 (EC driver)
%ISC
N.S.
Protrusion Formation
Cell Migration
D
w; tub::Gal80 ts, mira-GFP; pros::Gal4
Dsh RNAi Ecc15
E
w; how::Gal4/mira-GFP; tub::Gal80 ts
Control Ecc15
Otk RNAi Ecc15
%ISC
N.S.
Protrusion Formation
Cell Migration

Supplementary Figure 5: Controls for Dsh and Otk localization, and the effect of additional genetic perturbations in non-ISCs on migration, Related to Figure 3.

A. Staining for endogenously myc-tagged Dsh reveals cortical decoration in ISCs after 1hr post ablation. White arrows indicate Dsh+ ISCs and yellow arrows indicate Dsh- ISCs. B. Otk antibody co-localizes with GFP-tagged Otk expression. Data are representative of two independent experiments. C. Montage and quantification of ISC migration after overexpressing Otk in EBs and ECs. ISCs outlined in white, and montage tracks measured from the most distal region of the cell cortex. D. Montage of ISC migration after depleting Dsh in EEs. ISCs outlined in white, and montage tracks measured from the leading edge or, in cells that did not form protrusions, the most distal region of the cell cortex. E. Depleting Otk in the muscle layer did not significantly alter ISC migration. mean ± SD; n = sample size as follows, A: 7 flies from 2 (no ablation) and 3 (ablation) independent experiments, C: 6 (No Gal4, Mex1::Gal4) and 5 (SuH::Gal4) flies from 2 (No Gal4) and 3 (Mex1::Gal4, SuH::Gal4) independent experiments, E: 5 flies from 2 independent experiments. N.S. = not significant (C – Protrusion Formation: No Gal4 vs SuH::Gal4 = 0.7847, No Gal4 vs Mex1::Gal4 = 0.9839, C – Cell Migration: No Gal4 vs SuH::Gal4 = 0.8800, No Gal4 vs Mex1::Gal4 = 0.9269, E – Protrusion Formation = 0.5644, E – Cell Migration = 0.1298), ***P<0.001 (A<0.0001), based on two-tailed Student's t-test (A,E) and one-way ANOVA with Dunnett test (C). Scale bar = 10µm. Timestamp indicated as hours:minutes. Source data are provided as a Source Data file.

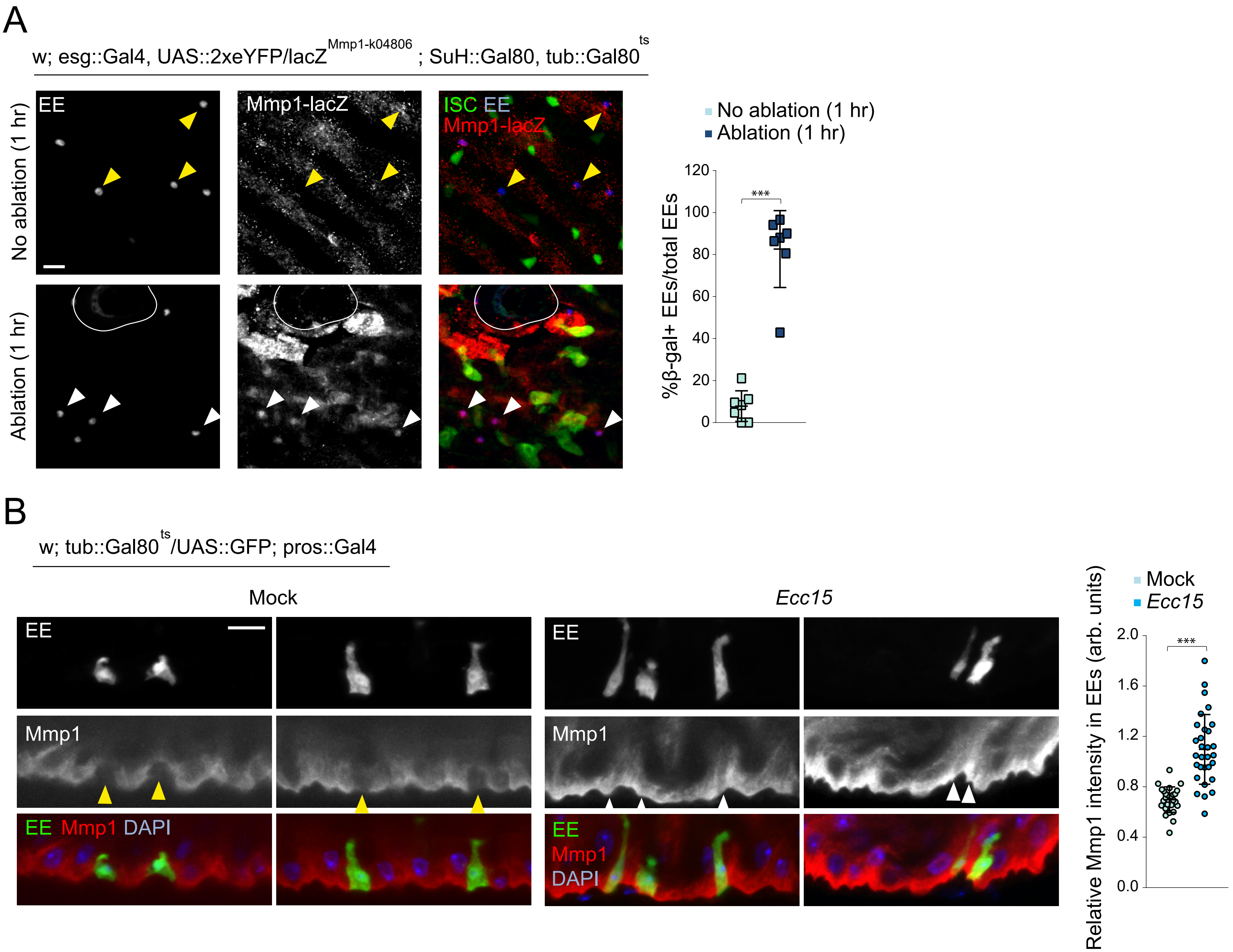
A
w; esg::Gal4, UAS::2xeYFP/lacZ Mmp1-k04806 ; SuH::Gal80, tub::Gal80 ts
No ablation (1 hr)
Ablation (1 hr)
EE
Mmp1-lacZ
ISC EE
Mmp1-lacZ
No ablation (1 hr)
Ablation (1 hr)
%β-gal+ EEs/total EEs
***
0
20
40
60
80
100
120
B
w; tub::Gal80 ts /UAS::GFP; pros::Gal4
Mock
Ecc15
EE
Mmp1
EE Mmp1 DAPI
Mock
Ecc15
Relative Mmp1 intensity in EEs (arb. units)
***
0.0
0.4
0.8
1.2
1.6
2.0

Supplementary Figure 6: Additional analyses of Mmp1 activity after tissue damage, Related to Figure 4.

A. Staining for beta Galactosidase in Mmp1-lacZ reporter lines reveal Mmp1 expression in EEs after 1hr post laser ablation. White arrowheads indicate β-gal+ EEs and yellow arrowheads indicate β-gal- EEs. White outline indicates ablation site. B. Mmp1 levels, as determined by immunostaining, increased in EEs after tissue damage. White arrowheads indicate EEs with high Mmp1 levels and yellow arrowheads indicate EEs with low Mmp1 levels. mean ± SD; n = sample size as follows, A: 7 flies from 3 (no ablation) and 2 (ablation) independent experiments, B: 27 cells from 7 flies (Mock) and 30 cells from 6 flies (*Ecc15*) from 2 independent experiments; ***P<0.001 (A,B<0.0001), based on two-tailed Student’s t-test. Scale bar = 10µm. Source data are provided as a Source Data file.

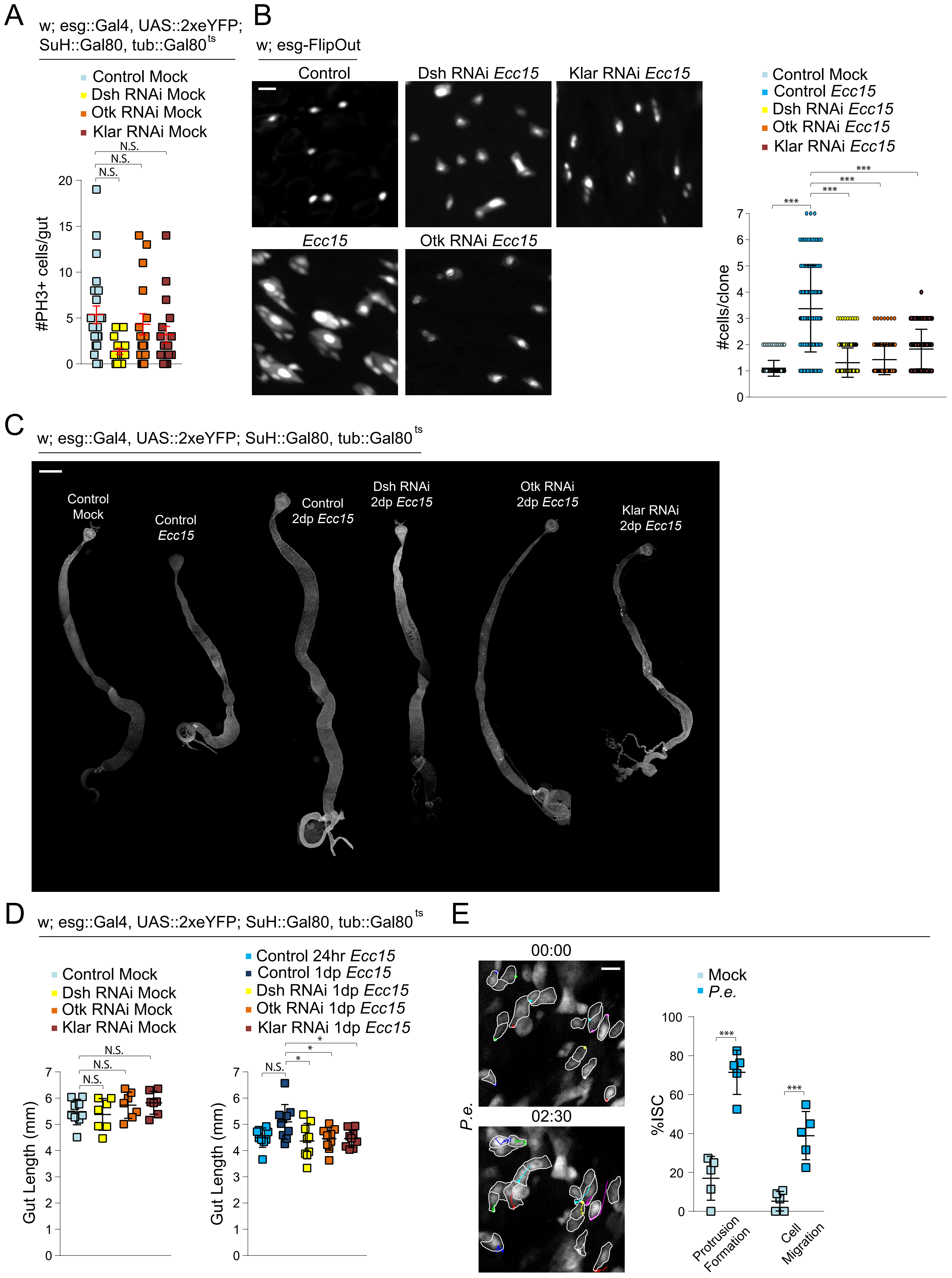
A
w; esg::Gal4, UAS::2xeYFP; SuH::Gal80, tub::Gal80ts
Control Mock
Dsh RNAi Mock
Otk RNAi Mock
Klar RNAi Mock
N.S.
#PH3+ cells/gut
B
w; esg-FlipOut
Control
Dsh RNAi Ecc15
Klar RNAi Ecc15
Ecc15
Otk RNAi Ecc15
Control Mock
Control Ecc15
Dsh RNAi Ecc15
Otk RNAi Ecc15
Klar RNAi Ecc15
#cells/clone
***
C
w; esg::Gal4, UAS::2xeYFP; SuH::Gal80, tub::Gal80ts
Control Mock
Control Ecc15
Control 2dp Ecc15
Dsh RNAi 2dp Ecc15
Otk RNAi 2dp Ecc15
Klar RNAi 2dp Ecc15
D
w; esg::Gal4, UAS::2xeYFP; SuH::Gal80, tub::Gal80ts
Control Mock
Dsh RNAi Mock
Otk RNAi Mock
Klar RNAi Mock
Control 24hr Ecc15
Control 1dp Ecc15
Dsh RNAi 1dp Ecc15
Otk RNAi 1dp Ecc15
Klar RNAi 1dp Ecc15
Gut Length (mm)
E
00:00
02:30
P.e.
Mock
P.e.
%ISC
Protrusion Formation
Cell Migration

Supplementary Figure 7: Additional effects of impairing ISC migration on tissue physiology, and migratory behavior after *P.e.* infection, Related to Figure 6.

A. Quantification of mitotic numbers after disrupting ISC migration in intestines under homeostatic conditions. Control mock data were taken from Figure 6A, as they served as genetic controls. B. esg-FlipOut clones increased in size after 24hr *Ecc15* infection. This increase in clone size was stunted when ISC migration was impaired. RNAi was induced for a total of four days, and flies were fed *Ecc15* 24hrs before dissection (during the final day of RNAi expression). C. Representative images of full-length intestines. D. Gut length after impairing ISC migration in homeostatic condition and one day of recovery after 24hr *Ecc15* infection. Controls for mock and 24hr *Ecc15* conditions were taken from Figure 6C. E. Montage and quantification of migratory behavior after *P.e.* infection. ISCs outlined in white, and montage tracks measured from the leading edge or, in cells that did not form protrusions, the most distal region of the cell cortex. Mock-treated controls were taken from Figure 1B. mean ± SEM (A) or mean ± SD (B,D,E); n = sample size as follows, A: 23 (Control), 16 (Dsh RNAi, Otk RNAi), and 17 (Klar RNAi) flies from 2 (all conditions except Otk RNAi) and 3 (Otk RNAi) independent experiments, B: 129 (Control Mock), 150 (Control *Ecc15*), 187 (Dsh RNAi), 174 (Otk RNAi), and 131(Klar RNAi) clones from 5 flies from 2 independent experiments, D: 9 (Control Mock, Control 24hr, Dsh RNAi 1dp, Otk RNAi 1dp, Klar RNAi 1dp), 7 (Dsh RNAi Mock, Otk RNAi Mock, Klar RNAi Mock), and 10 flies (Control 1dp) from 2 independent experiments, E: n=5 flies from 5 (Mock) and 2 (*P.e.*) independent experiments; N.S. = not significant (A: Control vs Dsh RNAi = 0.3606, Control vs Otk RNAi = 0.9845, Control vs Klar RNAi = 0.8818, D: Control Mock vs Dsh RNAi Mock = 0.9678, Control Mock vs Otk RNAi Mock = 0.6170, Control Mock vs Klar RNAi Mock = 0.3926, Control 24hr vs Control 1dp = 0.0506), *P<0.05 (D: Control 1dp vs Dsh RNAi 1dp = 0.0133, Control 1dp vs Otk RNAi 1dp = 0.0328, Control 1dp vs Klar RNAi 1dp = 0.0348), ***P<0.001 (B, E – Protrusion Formation < 0.0001, E – Cell Migration = 0.0005), based on one-way ANOVA with Dunnett test (A,B,D) and two-tailed Student's t-test (E). Scale bar = 10µm (B,E), 500µm (C). Timestamp indicated as hours:minutes. Source data are provided as a Source Data file.
