# Supplementary material for: Non-canonical Wnt signaling promotes directed migration of intestinal stem cells to sites of injury
Source: Nat Commun. 2021 Dec 9;12:7150. doi: 10.1038/s41467-021-27384-4 (PMC8660829; doi:10.1038/s41467-021-27384-4)
Supplement: Supplementary file 2 — Description of Additional Supplementary Files [file 41467_2021_27384_MOESM2_ESM.pdf]

Description of Additional Supplementary Files

File Name: Supplementary Data 1
Description: List of genotypes in each figure, Related to Methods

File Name: Supplementary Movie 1
Description: **ISC migratory behavior during homeostasis, Related to Figure 1. Cytoplasmic** GFP and mCherrytagged NLS was expressed in ISCs for four days. The fly was fed 5% sucrose for 16 hours, before the intestine dissected and imaged ex vivo at 10-min intervals. Time stamp indicated as hours:minutes:seconds. Video dimensions are 63µm x 63µm (width x height). Tracks measured from the center of the nucleus.

File Name: Supplementary Movie 2
Description: **ISC migratory behavior after Ecc15 infection, Related to Figure 1.** Cytoplasmic GFP and mCherrytagged NLS was expressed in ISCs for four days. The fly was infected with Ecc15 for 16 hours, before the intestine were dissected and imaged ex vivo at 10-min intervals. Time stamp indicated as hours:minutes:seconds. Video dimensions are 63µm x 63µm (width x height). Tracks measured from the center of the nucleus.

File Name: Supplementary Movie 3
Description: **EB migratory behavior after Ecc15 infection, Related to Figure 1. Cytoplasmic** GFP was expressed in EBs for four days. The fly was infected with Ecc15 for 16 hours, before the intestine was dissected and imaged ex vivo at 10-min intervals. Tracks were measured at the most distal region of the cell cortex. Time stamp indicated as hours:minutes:seconds. Video dimensions are 81µm x 81µm (width x height).

File Name: Supplementary Movie 4
Description: **EE migratory behavior after Ecc15 infection, Related to Figure 1**. Cytoplasmic GFP was expressed in EEs for four days. The fly was infected with Ecc15 for 16 hours, before the intestine was dissected and imaged ex vivo at 10-min intervals. Tracks were measured at the most distal region of the cell cortex. Time stamp indicated as hours:minutes:seconds. Video dimensions are 81µm x 81µm (width x height).

File Name: Supplementary Movie 5
Description: **ISC migratory behavior in undamaged intestine, Related to Figure 1.** Cytoplasmic GFP was expressed in ISCs for four days. Undamaged intestine was imaged ex vivo at 10-min intervals on the 2-photon microscopy system, and served as a control for ablated intestines. Minimal protrusion formations occurred. Time stamp indicated as hours:minutes:seconds. Video dimensions are 137µm x 137µm (width x height).

File Name: Supplementary Movie 6
Description: **ISC migratory behavior after ablation, Related to Figure 1.** Cytoplasmic GFP was expressed in ISCs for four days. Intestine was dissected and imaged ex vivo at 10-min intervals. Laser ablation occurred 30” before the second time point, resulting in multiple ISCs exhibiting protrusion formation throughout the remainder of the capture. Time stamp indicated as hours:minutes:seconds. Video dimensions are 137µm x 137µm (width x height).

File Name: Supplementary Movie 7

Description: **The actin cytoskeleton is largely static under homeostatic conditions, Related to Figure 1.** LifeAct-GFP and mCherry-tagged NLS was expressed in ISCs for four days. The fly was fed 5% sucrose for 16 hours, before the intestine was dissected, treated with 0.1% DMSO for 30�even, and imaged ex vivo at 10-min intervals. Time stamp indicated as hours:minutes:seconds. Video dimensions are 63µm x 63µm (width x height).

File Name: Supplementary Movie 8
Description: **Actin dynamics greatly increase after Ecc15 infection, Related to Figure 1**. LifeAct-GFP and mCherry-tagged NLS was expressed in ISCs for four days. The fly was infected with Ecc15 for 16 hours, before the intestine was dissected, treated with 0.1% DMSO for 30‟, and imaged ex vivo at 10-min intervals. Time stamp indicated as hours:minutes:seconds. Video dimensions are 63µm x 63µm (width x height).

File Name: Supplementary Movie 9
Description: **Treatment with Cytochalasin B abolishes actin dynamics in Ecc15-infected intestine, Related to Figure 1.** LifeAct-GFP was expressed in ISCs for four days. The fly was infected with Ecc15 for 16 hours, before the intestine was dissected, treated with 10µM Cytochalasin B for 30‟, and imaged ex vivo at 10-min intervals. Time stamp indicated as hours:minutes:seconds. Video dimensions are 33µm x 39µm (width x height).

File Name: Supplementary Movie 10
Description: **Arp3 RNAi impairs ISC migratory behavior in Ecc15-infected intestine, Related to Figure 1.** Arp3 RNAi and cytoplasmic GFP was expressed in ISCs for four days. The fly was infected with Ecc15 for 16 hours, before the intestine was dissected and imaged ex vivo at 10-min intervals. Tracks were measured from the edge of the protrusion, or, for ISCs that did not exhibit protrusion formation, the most distal region of the cell cortex. Time stamp indicated as hours:minutes:seconds. Video dimensions are 81µm x 81µm (width x height).

File Name: Supplementary Movie 11
Description: **Klar RNAi impairs cell body translocation but not protrusion formation in Ecc15- infected intestine, Related to Figure 1.** Klar RNAi and cytoplasmic GFP was expressed in ISCs for four days. The fly was infected with Ecc15 for 16 hours, before the intestine was dissected and imaged ex vivo at 10-min intervals. Tracks were measured from the edge of the protrusion, or, for ISCs that did not exhibit protrusion formation, the most distal region of the cell cortex. Time stamp indicated as hours:minutes:seconds. Video dimensions are 81µm x 81µm (width x height). Supplemental Video 1

File Name: Supplementary Movie 12
Description: **Dsh RNAi impairs ISC migratory behavior in Ecc15-infected intestine, Related to Figure 2.** Dsh RNAi and cytoplasmic GFP was expressed in ISCs for four days. The fly was infected with Ecc15 for 16 hours, before the intestine was dissected and imaged ex vivo at 10-min intervals. Tracks were measured from the edge of the protrusion, or, for ISCs that did not exhibit protrusion formation, the most distal region of the cell cortex. Time stamp indicated as hours:minutes:seconds. Video dimensions are 81µm x 81µm (width x height). Supplemental Video 1

File Name: Supplementary Movie 13
Description: **Otk RNAi impairs ISC migratory behavior in Ecc15-infected intestine, Related to Figure 2.** Otk RNAi and cytoplasmic GFP was expressed in ISCs for four days. The fly was infected with Ecc15 for 16 hours, before the intestine was dissected and imaged ex vivo at 10-min intervals. Tracks were measured

from the edge of the protrusion, or, for ISCs that did not exhibit protrusion formation, the most distal region of the cell cortex. Time stamp indicated as hours:minutes:seconds. Video dimensions are 81µm x 81µm (width x height).

File Name: Supplementary Movie 14
Description: **Dsh RNAi impairs ISC migratory behavior in ablated intestine, Related to Figure 2**. Dsh RNAi and cytoplasmic GFP was expressed in ISCs for four days. Intestine was dissected and imaged ex vivo at 10-min intervals. Laser ablation occurred 30" before the second time point. Time stamp indicated as hours:minutes:seconds. Video dimensions are 137µm x 137µm (width x height).

File Name: Supplementary Movie 15
Description: **Otk RNAi impairs ISC migratory behavior in Ecc15-infected intestine, Related to Figure 2.** Otk RNAi and cytoplasmic GFP was expressed in ISCs for four days. Intestine was dissected and imaged ex vivo at 10-min intervals. Laser ablation occurred 30" before the second time point. Time stamp indicated as hours:minutes:seconds. Video dimensions are 137µm x 137µm (width x height).

File Name: Supplementary Movie 16
Description: **Treatment with LGK974 abolishes ISC migratory behavior in ablated intestine, Related to Figure 2.** Cytoplasmic GFP was expressed in ISCs for four days. Intestine was dissected, treated with 1µM LGK974 B for 30‟, and imaged ex vivo at 10-min intervals. Laser ablation occurred 30" before the second time point. Time stamp indicated as hours:minutes:seconds. Video dimensions are 137µm x 137µm (width x height).

File Name: Supplementary Movie 17
Description: **Otk OE promotes protrusion dynamics, Related to Figure 2.** Otk-GFP and cytoplasmic GFP was expressed in ISCs for four days. Intestine was dissected and imaged ex vivo at 10-min intervals. Time stamp indicated as hours:minutes:seconds. Video dimensions are 39µm x 33µm (width x height).

File Name: Supplementary Movie 18
Description: **Otk OE in EEs promotes migratory behavior in ISCs, Related to Figure 3.** Otk was overexpressed in EEs for four days, and ISCs were specifically labelled with miraGFP. Intestine was dissected and imaged ex vivo at 10-min intervals. Tracks were measured from the edge of the protrusion, or, for ISCs that did not exhibit protrusion formation, the most distal region of the cell cortex. Time stamp indicated as hours:minutes:seconds. Video dimensions are 81µm x 81µm (width x height).

File Name: Supplementary Movie 19
Description: **Otk RNAi in EEs impairs migratory behavior in ISCs in Ecc15- infected intestine, Related to Figure 3.** Otk RNAi was expressed in EEs for four days, and ISCs were specifically labelled with miraGFP. The fly was infected with Ecc15 for 16 hours, before the intestine was dissected and imaged ex vivo at 10-min intervals. Tracks were measured from the edge of the protrusion, or, for ISCs that did not exhibit protrusion formation, the most distal region of the cell cortex. Time stamp indicated as hours:minutes:seconds. Video dimensions are 81µm x 81µm (width x height).

File Name: Supplementary Movie 20
Description: **Treatment with Ptk7 promotes ISC migratory, Related to Figure 4.** Lifeact-GFP was expressed in ISCs for four days. Intestine was dissected, treated with 1µg/ml Nterminal Ptk7 for 30‟, and

imaged ex vivo at 10-min intervals. Time stamp indicated as hours:minutes:seconds. Video dimensions are 39µm x 33µm (width x height).

File Name: Supplementary Movie 21
Description: **Otk RNAi abolishes protrusion formation in Ptk7-treated intestine, Related to Figure 4.** Otk RNAi and Lifeact-GFP was expressed in ISCs for four days. Intestine was dissected, treated with 1µg/ml N-terminal Ptk7 for 30‟, and imaged ex vivo at 10- min intervals. Time stamp indicated as hours:minutes:seconds. Video dimensions are 39µm x 33µm (width x height).

File Name: Supplementary Movie 22
Description: **Timp OE in EEs impairs ISC migratory behavior in Ecc15- infected intestine, Related to Figure 4.** Timp was overexpressed in EEs for four days, and ISCs were specifically labelled with miraGFP. The fly was infected with Ecc15 for 16 hours, before the intestine was dissected and imaged ex vivo at 10-min intervals. Time stamp indicated as hours:minutes:seconds. Video dimensions are 81µm x 81µm (width x height).

File Name: Supplementary Movie 23
Description: **Treatment with TAPI-1 abolishes ISC migratory behavior in ablated intestine, Related to Figure 4.** Cytoplasmic GFP was expressed in ISCs for four days. Intestine was dissected, treated with 20µM TAPI-1 for 30‟, and imaged ex vivo at 10-min intervals. Laser ablation occurred 30” before the second time point. Time stamp indicated as hours:minutes:seconds. Video dimensions are 137µm x 137µm (width x height).

File Name: Supplementary Movie 24
Description: **Wee1 OE impairs ISC migratory behavior in Ecc15-infected intestine, Related to Figure 6.** Wee1 and cytoplasmic GFP was overexpressed in ISCs for four days. The fly was infected with Ecc15 for 16 hours, before the intestine was dissected and imaged ex vivo at 10-min intervals. Tracks were measured from the edge of the protrusion, or, for ISCs that did not exhibit protrusion formation, the most distal region of the cell cortex. Time stamp indicated as hours:minutes:seconds. Video dimensions are 81µm x 81µm (width x height).

File Name: Supplementary Movie 25
Description: **Wee1 RNAi promotes ISC migratory behavior, Related to Figure 6**. Wee1 RNAi and cytoplasmic GFP was expressed in ISCs for four days. The intestine was dissected and imaged ex vivo at 10-min intervals. Tracks were measured from the edge of the protrusion, or, for ISCs that did not exhibit protrusion formation, the most distal region of the cell cortex. Time stamp indicated as hours:minutes:seconds. Video dimensions are 81µm x 81µm (width x height).
