# Supplementary material for: Non-canonical Wnt signaling promotes directed migration of intestinal stem cells to sites of injury
Source: Nat Commun. 2021 Dec 9;12:7150. doi: 10.1038/s41467-021-27384-4 (PMC8660829; doi:10.1038/s41467-021-27384-4)
Supplement: Supplementary file 29 — Reporting Summary [file 41467_2021_27384_MOESM29_ESM.pdf]

nature portfolio

Corresponding author(s): Heinrich Jasper

Last updated by author(s): Oct 22, 2021

Reporting Summary

Nature Portfolio wishes to improve the reproducibility of the work that we publish. This form provides structure for consistency and transparency in reporting. For further information on Nature Portfolio policies, see our Editorial Policies and the Editorial Policy Checklist.

Statistics

For all statistical analyses, confirm that the following items are present in the figure legend, table legend, main text, or Methods section.

n/a	Confirmed	
☐	☒	The exact sample size (*n*) for each experimental group/condition, given as a discrete number and unit of measurement
☐	☒	A statement on whether measurements were taken from distinct samples or whether the same sample was measured repeatedly
☐	☒	The statistical test(s) used AND whether they are one- or two-sided<br>*Only common tests should be described solely by name; describe more complex techniques in the Methods section.*
☒	☐	A description of all covariates tested
☐	☒	A description of any assumptions or corrections, such as tests of normality and adjustment for multiple comparisons
☐	☒	A full description of the statistical parameters including central tendency (e.g. means) or other basic estimates (e.g. regression coefficient) AND variation (e.g. standard deviation) or associated estimates of uncertainty (e.g. confidence intervals)
☐	☒	For null hypothesis testing, the test statistic (e.g. *F*, *t*, *r*) with confidence intervals, effect sizes, degrees of freedom and *P* value noted<br>*Give P values as exact values whenever suitable.*
☒	☐	For Bayesian analysis, information on the choice of priors and Markov chain Monte Carlo settings
☒	☐	For hierarchical and complex designs, identification of the appropriate level for tests and full reporting of outcomes
☒	☐	Estimates of effect sizes (e.g. Cohen's *d*, Pearson's *r*), indicating how they were calculated

Our web collection on statistics for biologists contains articles on many of the points above.

Software and code

Policy information about availability of computer code

Data collection	No software was used.
Data analysis	Data was analyzed with GraphPad Prism.

For manuscripts utilizing custom algorithms or software that are central to the research but not yet described in published literature, software must be made available to editors and reviewers. We strongly encourage code deposition in a community repository (e.g. GitHub). See the Nature Portfolio guidelines for submitting code & software for further information.

Data

Policy information about availability of data

All manuscripts must include a data availability statement. This statement should provide the following information, where applicable:

- Accession codes, unique identifiers, or web links for publicly available datasets
- A description of any restrictions on data availability
- For clinical datasets or third party data, please ensure that the statement adheres to our policy

Data sharing not applicable to this article as no datasets were generated or analyzed during the current study.

Field-specific reporting

Life sciences study design

All studies must disclose on these points even when the disclosure is negative.

Sample size	No statistical methods were used to calculate the sample size. Sample numbers are described in the corresponding figure legends. Sample sizes were determined according to our previous publications (i.e. https://doi.org/10.1038/s41467-019-08982-9 and https://doi.org/10.1016/j.celrep.2019.08.014) and literature in the field (i.e. https://doi.org/10.1016/j.cub.2020.01.082), as we are using similar experimental paradigms. 3 guts were frequently used as a minimum sample size for each condition in these paradigms, and we increased the minimum sample size to 5 guts in this study.
Data exclusions	No data was excluded in this study.
Replication	All the experiments were repeated at least twice, as specified in the Methods section. All repeats were successful.
Randomization	For comparison of flies of the same cohort (i.e. genotype) with different treatments, female 10d flies were randomly assigned to control or experimental groups. Dissected intestines were selected randomly from flies of the same cohort. The specific region imaged was chosen at random within R4 of the posterior midgut.
Blinding	All experiments were quantified blindly without knowledge of genotype and/or treatment.

Reporting for specific materials, systems and methods

We require information from authors about some types of materials, experimental systems and methods used in many studies. Here, indicate whether each material, system or method listed is relevant to your study. If you are not sure if a list item applies to your research, read the appropriate section before selecting a response.

Materials & experimental systems

n/a	Involved in the study	
☐	☒	Antibodies
☒	☐	Eukaryotic cell lines
☒	☐	Palaeontology and archaeology
☐	☒	Animals and other organisms
☒	☐	Human research participants
☒	☐	Clinical data
☒	☐	Dual use research of concern

Methods

n/a	Involved in the study	
☒	☐	ChIP-seq
☒	☐	Flow cytometry
☒	☐	MRI-based neuroimaging

Antibodies

Antibodies used	Anti mouse: Prospero (1:300, DHSB, clone name: MR1A, catalog no: Prospero (MR1A), RRID:AB_528440), Myc (1:300, Abcam, clone name: 9E10, catalog no: ab32, RRID:AB_303599), and Mmp1 (1:10, DHSB, clone name: 5H7B11, catalog no: 5H7B11, RRID: AB_579779; clone name: 3A6B4, catalog no: 3A6B4, RRID:AB_579780; clone name: 3B8D12, catalog no: 3B8D12, RRID: AB_579781). Anti rabbit: ß-galactosidase (MP Biomedicals, 085597-CF), phospho-Histone H3 (1:1000, EMD Millipore, 06-570, RRID:AB_310177), C-terminal Otk (1:50), and N-terminal Otk (1:50). Anti chicken: GFP (1:300, Abcam, ab13970, RRID:AB_300798). Secondary cyanine dye antibodies (1:300, Jackson ImmunoResearch Laboratories).
Validation	Generated antibodies for Otk were validated by immunostaining, demonstrating complete co-localization with expression of exogenous GFP-tagged Otk, as provided in data from the manuscript (Fig. 6a and Supplemental Fig. 5b). Validation for other antibodies was done previously by others, as these antibodies are commonly used and published antibodies for immunostaining. Each antibody has been cited at least a dozen times and validated to work with immunostaining, as listed in the manufacturer's website.

Animals and other organisms

Policy information about studies involving animals; ARRIVE guidelines recommended for reporting animal research

Laboratory animals	10-11d female Drosophila melanogaster were used for this study. Genotypes used in each experiment are detailed in Supplemental Table 1.
Wild animals	No wild animals were used in this study.
Field-collected samples	No field-collected samples were used in this study.
Ethics oversight	This study did not require ethics approval.

Note that full information on the approval of the study protocol must also be provided in the manuscript.

March 2021
